# A Closed‐Loop Recyclable Low‐Density Polyethylene

**DOI:** 10.1002/advs.202307229

**Published:** 2024-01-23

**Authors:** Christoph Unger, Holger Schmalz, Jannis Lipp, Winfried P. Kretschmer, Rhett Kempe

**Affiliations:** ^1^ Anorganische Chemie II – Katalysatordesign Sustainable Chemistry Centre Universität Bayreuth Universitätsstraße 30 NW I D‐95440 Bayreuth Germany; ^2^ Makromolekulare Chemie II, Bavarian Polymer Institute (BPI) Universität Bayreuth Universitätsstraße 30 NW I D‐95440 Bayreuth Germany

**Keywords:** circular economy, closed‐loop recycling, coordinative chain transfer polymerization, LDPE, polyethylene

## Abstract

Low‐density polyethylene (LDPE) is one of the most important plastics, which is produced unfortunately under extreme conditions. In addition, it consists of robust aliphatic C─C bonds which are challenging to cleave for plastic recycling. A low‐pressure and ‐temperature (p_ethylene_ = 2 bara, T = 70 °C) macromonomer‐based synthesis of long chain branched polyethylene is reported. The introduction of recycle points permits the polymerization (grafting to) of the macromonomers to form the long chain branched polyethylene and its depolymerization (branch cleavage). Coordinative chain transfer polymerization employing ethylene and co‐monomers is used for the synthesis of the macromonomers, permitting a high flexibility of their precise structure and efficient synthesis. The long chain branched polyethylene material matches key properties of low‐density polyethylene.

## Graphical abstract

Controlled and efficient ethylene copolymerization via coordinative chain transfer polymerization permits the synthesis of low density polyethylene materials that contain recycle points. The syntheses proceed under mild conditions and the recycle points permit closed‐loop recycling of the plastic.

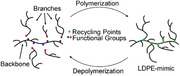

## 1 Introduction

**Figure 1:**
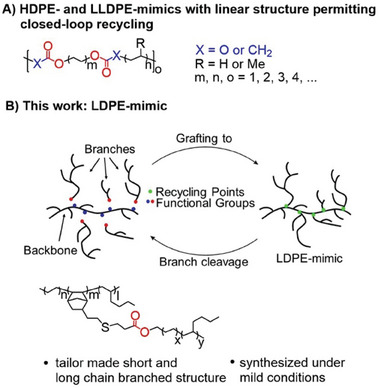
State‐of‐the‐art regarding polyethylene mimics and the work presented here. A) High‐density polyethylene (HDPE) mimic or linear low‐density polyethylene (LLDPE) mimic based on bio‐based, propylene‐ or (poly)ethylene‐based feedstock. B) Structural and functional low‐density polyethylene (LDPE) mimic with recycle points introduced here.

Low‐density polyethylene (LDPE) is one of the most important plastics used daily by nearly everybody due to its numerous applications as packing material. It is produced via a radical process under extremely high pressure (≈2000 bar) and at elevated temperatures.^[^ ^1^ ^]^ Unfortunately, the unique structure of this polyethylene material^[^ ^2^ ^]^ is challenging to synthesize with polymerization protocols that operate under significantly milder conditions.^[^ ^3^, ^4^ ^]^ Polyethylene mimics permitting closed‐loop recycling have recently been disclosed, such as mimics of high‐density polyethylene using bio‐based macromonomers^[^ ^5^, ^6^, ^7^ ^]^ or polyethylene/ethylene‐based macromonomers^[^ ^8^, ^9^ ^]^ and a mimic of LLDPE employing propylene as feedstock^[^ ^10^ ^]^ (**Figure** **1**A). All these polyethylene mimics have a linear structure and ester or carbonate linkages permit the polymerization and depolymerization of the macromonomers. Coordinative chain transfer polymerization (CCTP),^[^ ^11^ ^]^ a synthesis protocol that permits the controlled and efficient polymerization of ethylene and related monomers,^[^ ^12^, ^13^, ^14^ ^]^ also with very high catalyst economy,^[^ ^15^ ^]^ could be a suitable protocol for synthesis tailor‐made macromonomers to structurally mimic LDPE. Ethylene an attractive and also green or sustainable feedstock, if synthesized from ethanol,^[^ ^16^ ^]^ is the main starting material then. Herein we report on a low‐pressure and ‐temperature (p_ethylene_ = 2 bara, T = 70 °C) macromonomer‐based synthesis of long chain branched polyethylene (Figure 1B). The introduction of recycle points (ester linkages) permits grafting and degrafting of the macromonomers via acidic esterification and basic saponification. The two components (backbone and branch) are low molecular weight macromonomers, which are well soluble in a variety of organic solvents for separation from other polymers such as HDPE or *i*PP. The CCTP of ethylene and co‐monomers is used for the synthesis of the functionalized macromonomers permitting a high flexibility of their precise structure and economic or efficient synthesis. Our long chain branched polyethylene material matches the key properties of LDPE.

## 2 Results and Discussion

**Figure 2:**
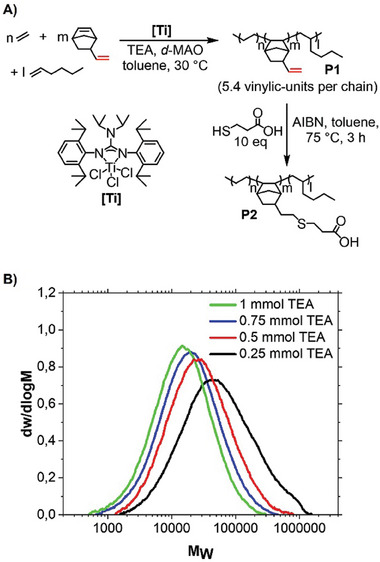
Synthesis of the backbone P2. A) Synthesis of the unsaturated polymer backbone P1 by terpolymerization of ethylene, 1‐hexene, and vinylnorbornene (VNB) with titanium precatalyst [Ti] and functionalization by thiol‐ene click chemistry to an acid‐functionalized backbone P2 (AIBN = azobisisobutyronitrile). B) Gel permeation chromatography (GPC) traces of the ethylene/1‐hexene/VNB terpolymers synthesized by different triethylaluminium (TEA) concentrations show the flexibility in the synthesis of the backbone chain length.

First, we became interested in the synthesis of the backbone or the polyethylene grafting support. Irreversible CCTP employing a titanium catalyst [Ti], developed in our group,^[^ ^17^ ^]^ was used to combine efficiency and control in the terpolymerization of ethylene, 1‐hexene, and vinylnorbornene (VNB) (**Figure** **2**). The free double bonds introduced via VNB incorporation allow subsequent post‐functionalization chemistry. The ^1^H‐NMR analysis of these terpolymers shows the selective incorporation of the norbornene double bond, with negligible incorporation rates of the corresponding vinyl units. Crosslinking of the polymer chains as a side reaction was not observed. We varied the concentration of triethylaluminium during the polymerization process to synthesize short chain branched terpolymers of different molecular weights and demonstrate the flexibility of our backbone synthesis. Our terpolymers showed a high VNB incorporation of up to about eight per chain combined with good solubility in various organic solvents due to a high 1‐hexene incorporation – up to ≈18 per chain. Terpolymer synthesized by the conditions of Entry 2, **Table** **1** were used for backbone synthesis since a typical LDPE chain is characterized by three or more long chain branches.^[^ ^2^ ^]^ In addition, the lowest possible molecular weights were selected to obtain well‐soluble macromonomers.

**Table 1:** Terpolymerisation of ethylen/1‐hexene/VNB for the synthesis of the unsaturated polymer backbone P1.

entry^a)^	n_TEA_ [mmol]	${\bar{M}}_{n}$ [g/mol]	Ð	activity^b)^ $\left[\frac{{\mathrm{kg}}_{\mathrm{PE}}}{{\mathrm{mol}}_{\mathrm{cat}}\;h\;\mathrm{bar}}\right]$	average vinylicunits per chain^c)^	average 1‐hexene units per chain^c)^
1	0.25	24 200	4.3	1 730	8.8	18.3
2	0.50	14 900	3.1	1 420	5.4	11.3
3	0.75	10 400	3.0	1 200	3.8	7.90
4	1.00	8 000	2.8	1 080	2.9	6.10

^a)^ Reactants and conditions are as follows: n_VNB_ = 20 mmol, n_1‐hexene_ = 100 mmol, V_ethylene_ (converted) = 6 L_n_, V_toluene/VNB/1‐hexene_ = 75 mL, T = 30 °C, p_ethylene_ = 2.0 bara, n**_[Ti]_** = 6 µmol, n*_d_*_‐MAO_ = 3 mmol;

^b)^ Activity of the catalyst is based on the reaction time to consume 6 L_n_ ethylene (Figure S1, Supporting information);

^c)^ Incorporation of the average comonomer units per chain is calculated by GPC (M_n_) and ^13^C‐NMR (Supporting information).

We used thiol‐ene click chemistry for the conversion of the unsaturated terpolymer **P1** into the final backbone **P2**, as it can be considered as a simple tool for nearly quantitative functionalization of an olefin unit into a carboxylic acid.^[^ ^18^ ^]^ Thioether linkers also show a high stability under acidic and basic conditions,^[^ ^19^ ^]^ which is important for our grafting to and branch cleavage steps (*vide infra*). We reacted our unsaturated terpolymer **P1** with an excess of 3‐mercaptopropionic acid in the presence of azobisiso‐butyronitrile as a radical starter to yield the acid‐functionalized short chain branched polymer backbone **P2** within nearly quantitative conversion of the vinylic units.

**Figure 3:**
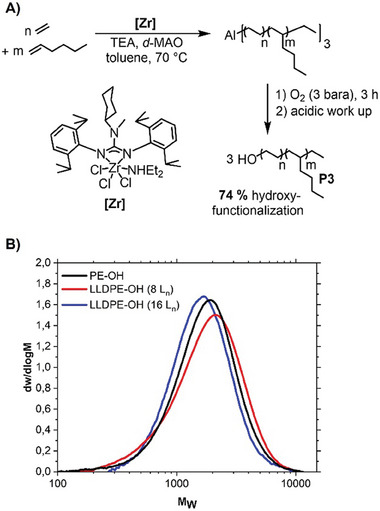
Synthesis of branches P3. A) One‐pot synthesis of the hydroxy‐terminated poly(ethylene‐*co*‐1‐hexene) branches by coordinative chain transfer polymerization with zirconium precatalyst [Zr] and oxidation with O_2_. B) GPC traces of the long‐chain alcohols P3.

We next became interested in the synthesis of the branches **P3** or the macro grafting agent. We recently reported a highly active CCTP zirconium catalyst [Zr] family with a very high catalyst economy^[^ ^15^ ^]^ and used it for the synthesis of the branches **P3** (**Figure** **3**). We copolymerized ethylene and 1‐hexene to yield **P3** (Entry 2–3, **Table** **2**). We aimed for a chain length between 1000 and 2000 g mol^−1^ since this is the proposed molecular weight of the branches of classic LDPE materials.^[^ ^2^ ^]^ Results of the copolymerization of ethylene and 1‐hexene are shown in Figure 3. We observed a very high activity and an average of one branch per macromolecule. After the conversion of the ethylene amount desired to reach the molecular weight targeted, we oxidized the polymer chain ends resting on the aluminum with pure oxygen and yielded linear, narrowly distributed, ethylene/1‐hexene copolymer alcohols. The ^1^H‐NMR analysis of the ethylene homopolymer indicates ≈74% hydroxy‐functionalization for the polymer chain length chosen here (Entry 5, Table 2).

**Table 2:** (Co)polymerization of ethylene(/1‐hexene) for the synthesis of the long chain alcohols P3.

entry^a)^	n_TEA_ [mmol]	n_1‐hexene_ [mmol]	V_eth_ [L_n_]	${\bar{M}}_{n}$ [g/mol]	Ð	activity^b)^ $\left[\frac{{\mathrm{kg}}_{\mathrm{PE}}}{{\mathrm{mol}}_{\mathrm{cat}}\;h\;\mathrm{bar}}\right]$	average 1‐hexene units per chain^c)^
5	2.5	/	8	1 360	1.5	13 800	/
6	2.5	100	8	1 370	1.6	4 000	1.0
7	5.0	100	16	1 350	1.4	4 100	1.0

^a)^ Reactants and conditions are as follows: n_1‐hexene_ = 100 mmol, V_toluene/1‐hexene_ = 180 mL, T = 70 °C, p_ethylene_ = 2.0 bara, n**_[Zr]_** = 1 µmol, n*_d_*_‐MAO_ = 0.5 mmol, p_O2_ = 3 bara, t_oxidation_ = 3 h. Degree of hydroxy‐functionalization determined for entry 5 by ^1^H‐NMR spectroscopy;

^b)^ Activity of the catalyst is based on the reaction time to consume the desired amount of ethylene (Figure S2, Supporting Information);

^c)^ Incorporation of the average 1‐hexene units per chain is calculated by GPC (M_n_) and ^13^C‐NMR (Supporting Information).

**Figure 4:**
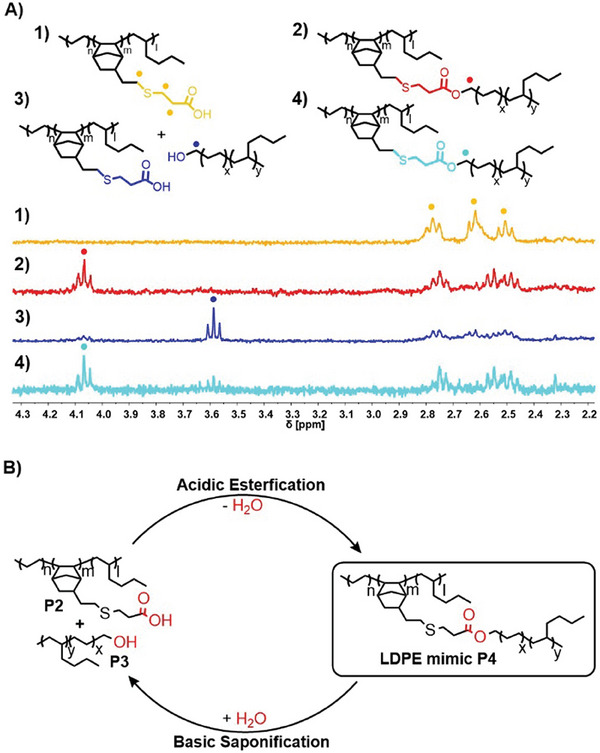
Grafting to and branch cleavage of the LDPE mimic P4. A) The ^1^H‐NMR stack of the carboxylic acid‐functionalized macromonomer P2 (yellow), LPDE mimic P4 (red), degrafted P4 (blue), and regrafted P4 (cyan), measured in C_2_D_2_Cl_4_ at 120 °C. B) Recycling cycle of the LDPE mimic. The linking of the two macromonomers P2 and P3 is performed under acidic conditions. The degrafting of the LDPE mimic P4 is conducted under basic conditions.

The linking of the two macromonomers **P2** and **P3** (**Figure** **4**) was conducted under simple acidic conditions (esterification). After 18 h, we obtained nearly quantitative grafting of the hydroxy‐functionalized branches **P3** to our backbone **P2**, resulting in the new LDPE mimic **P4**. The ^1^H‐NMR investigation of **P4** revealed ester‐based recycle points. The ester linkages can be cleaved under basic conditions in aqueous media (saponification of esters) to recover our macromonomers **P2** and **P3**. Almost quantitative cleavage of these ester groups was achieved within several hours by treating the LPDE mimic **P4** with 1 N NaOH above the melting point of the material at 110 °C in a two‐phase system. We converted the resulting depolymerized polymer blend of **P2** and **P3** again by acid‐catalyzed condensation under the original synthesis conditions (Figure 4) into our LDPE mimic to complete the recycling process.

**Figure 5:**
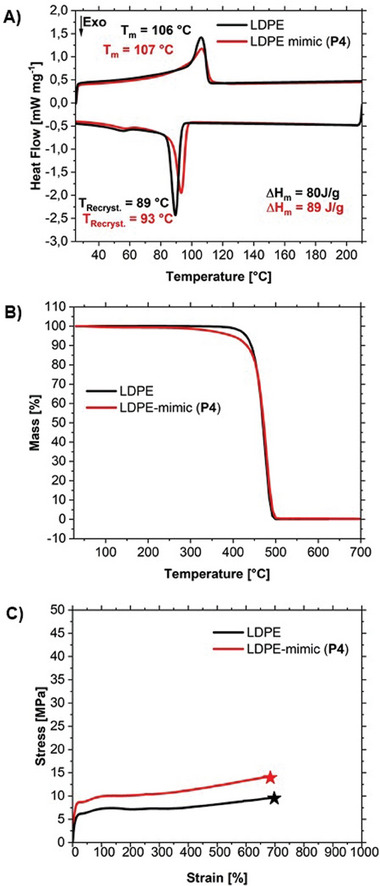
Characterization of our LDPE mimic and comparisons with a commercial LDPE material. A) DSC (second heating and cooling curve) of LDPE mimic P4 and commercial LDPE. B) TGA curve of P4 compared to commercial LDPE. C) Representative stress–strain curves for LDPE mimic P4 and LDPE.

The conversion of functional groups during the recycling cycle was confirmed by ^1^H‐NMR analysis, paying particular attention to the appearance and disappearance of ester groups (Figure 4A). Grafting to and branch cleavage are not quantitative. The thermal and mechanical properties of our LDPE mimic **P4** were studied by differential scanning calorimetry (DSC), thermogravimetric analysis (TGA), and tensile test and compared with a commercial LDPE (**Figure** **5**A–C). The commercial Lupolen LDPE 1800P was used for comparison. It is characterized by a melting point ≈106 °C and a density of 0.92 g cm^−3^, which is very similar to that of our LDPE mimic **P4** (107 °C, 0.93 g cm^−3^). It is also noticeable that the crystallization temperature of the commercial LDPE and **P4** is nearly identical. The TGA of the LDPE mimic **P4** and the commercial LDPE also show similar behavior. Obviously, the introduction of the recycle points and thioether linkers do not lead to a much earlier thermal decomposition. We also tested our highly branched polyethylene material in a tensile test and compared it with commercial LDPE. The tensile elongation behavior of our LDPE mimic **P4** is similar to commercial LDPE and shows a similar elongation at break, with a slightly different tensile strength. TGA and tensile tests of the recycled polymer are close to the data of the original polymer. Significant cross‐linking of our mimic is observed at 170 °C as reported.^[^ ^20^ ^]^

## 3 Conclusion

In conclusion, we introduced a new chemically recyclable polyolefin material. It consists of two different macromonomers, a backbone **P2** and potential long chain branches **P3**. The branches can be reversibly grafted to the backbone and cleaved under acidic and basic conditions. The synthesis of the two macromonomers is olefin‐based, highly flexible, and proceeds under mild conditions. The structure and properties of the final plastic material match the key properties of LDPE. Designing a single‐component macromonomer could be an improvement and avoiding the use of 3‐mercaptopropionic acid.

## Conflict of Interest

The authors declare no conflict of interest.

## Supporting information

Supporting Information

## Data Availability

The data that support the findings of this study are available in the supplementary material of this article.
