# Supplementary material for: A Closed‐Loop Recyclable Low‐Density Polyethylene
Source: Adv Sci (Weinh). 2024 Jan 23;11(13):2307229. doi: 10.1002/advs.202307229 (PMC10987147; doi:10.1002/advs.202307229)
Supplement: Supplementary file 1 — Supporting Information [file ADVS-11-2307229-s001.pdf]

ADVANCED SCIENCE
Open Access

Supporting Information

for *Adv. Sci.*, DOI 10.1002/advs.202307229

A Closed-Loop Recyclable Low-Density Polyethylene

*Christoph Unger, Holger Schmalz, Jannis Lipp, Winfried P. Kretschmer and Rhett Kempe**

Supporting Information
©Wiley-VCH 2021
69451 Weinheim, Germany

A closed-loop recyclable low-density polyethylene

C. Unger, H. Schmalz, J. Lipp, W. P. Kretschmer and R. Kempe*

SUPPORTING INFORMATION

Table of Contents

Materials and Methods 3
Synthetic Procedures....5
Ethylene Flow 7
NMR Spectra 8
GPC....19
DSC 22
TGA 24
Tensile Tests 26
Density Measurements 29
Rheology Measurements....30
Solubility Experiments 33
References 33

Materials and Methods

General Considerations

All manipulations of air and/or moisture sensitive compounds were performed under exclusion of oxygen by using standard Schlenk line techniques or an argon or nitrogen filled glove box (mBraun) with a high capacity circulator (< 0.1 ppm O_2).

Chemicals

Toluene (Sigma Aldrich, anhydrous, 99.8 %) and ethylene (3.5, Linde AG) used for polymerization experiments were passed over columns of R3-11 supported Cu oxygen scavenger (BASF) and Al_2O_3 (Fischer Scientific). Triethyl aluminum ($AlEt_3$, TEA) (SASOL Germany GmbH), 3-mercaptopropionicacid (Sigma Aldrich), 2,2′-azobis(2-methylpropionitrile) (AIBN) (Sigma Aldrich), tetra-*n*-butylammonium bromide (Alfa), HCl (Kraft), sodium hydroxide pellets (Sigma Aldrich) and ethanol *p.a.* (Sigma Aldrich) were used without further purification. 1-Hexene (Acros) and 5-vinyl-2-norbornene (VNB, Sigma Aldrich) were degassed, stirred over $LiAlH_4$ for 12 hours at room temperature, transferred under vacuum and stored under argon prior to usage. The *p*-toluenesulfonic acid (*p*-TosOH) monohydrate (Lancaster) was used as purchased. The *d*-MAO (depleted) was obtained by removing the volatile components from a methylaluminoxane solution in toluene (Chemtura Europe Limited). Commercial Lupolen LDPE 1800P was purchased from LyondellBasell.

Characterization Methods

^{1}H and ^{13}C NMR - Spectra were performed at a Varian INOVA 300 (^{1}H: 299.9 MHz, ^{13}C: 75.4 MHz). The ^{1}H- and ^{13}C-NMR polymer spectra were recorded at 120 °C in $C_2D_2Cl_4$ and referenced internally to the residual solvent resonances (^{1}H-NMR: 5.90 ppm; ^{13}C-NMR: 74.12 ppm). The ^{1}H-NMR spectra of the low molecular weight compounds were recorded at 25 °C in C_6D_6 and referenced also internally to the residual solvent resonances (^{1}H-NMR: 7.16 ppm). For the ^{13}C-NMR measurements 10 000 number of transitions and a relaxation delay of 9 s was used. Chemical shifts (δ) are reported in ppm. Mestre Nova software was used to evaluate the reported NMR spectra.

Gel Permeation Chromatography (GPC) - Analyses were carried out at an Agilent (Polymer Laboratories Ltd.) PL-GPC 220 high temperature chromatographic unit equipped with refractive index and differential pressure detectors, a guard and three linear mixed bed columns (Agilent Olexis). GPC analyses were performed at 150 °C using 1,2,4-trichlorobenzene as mobile phase. The samples were prepared by dissolving the polymer (0.1 wt.-%) in the mobile phase in an external oven and were run without filtration. The molecular weights of the samples were referenced to linear narrow HDPE standards (M_w = 110 – 430 000 g/mol, K = 40.6 and α = 0.725)

Differential Scanning Calorimetry (DSC) - DSC measurements were performed on a Mettler DSC 3+. Standard aluminum pans with a volume of 40 µl were used. The samples were measured in two cycles from 20 to 210 °C, a heating rate of 10 K/min and under a nitrogen atmosphere. The T_m and $T_{recryst.}$ was taken from the second heating/cooling curve.

Thermogravimetric Analysis (TGA) - All TGA measurements were performed on a Mettler Toledo TGA / SDTA 851e. Standard aluminum pans with a volume of 40 µL were used. Samples were measured from 20 °C to 700 °C with a heating rate of 10 K/min under a nitrogen atmosphere.

Compression Molding - Compression molding for dogbone-shaped samples was conducted at a Carver Typ 2518 hotpress. Films around 0.5 mm thickness were compression molded at 115 °C or 120 °C for 4 minutes at a pressure of 1 ton. All polymer processing was performed without the addition of additives (stabilizers or antioxidants).

Density Measurements – The density measurements were performed on a Mettler-Toledo weight using the Archimedes principle. Five compression molded samples (30 mg - 50 mg) were weighed once on the air and the other time the weight of the sample completely immersed in water (T = 22.4 °C, ρ = 0.9977 g/cm^3) was measured.

Tensile Elongation Tests – The tensile tests were performed on an Instron universal testing machine 5565, equipped with a video extensometer using dogbone-shaped samples [0.5 mm (T) x 4 mm (W) x 15 mm (L)]. Tensile testing was performed at a ramp speed of 10 mm/min. Six replicates per material were used for the elongation to break tests.

Rheology Measurements – The rheology measurements were conducted on a plate rheometer (Anton Paar MCR 302). Rheology plates (thickness = 1 mm, diameter = 25 mm) were previously compression molded on a Carver Typ 2518 hotpress at 120 °C for 6 minutes at a pressure of 1 ton. The polymer processing for synthesized polymer samples was performed with the addition of commercially available *Irganox 1010* as stabilizer. The viscoelastic region was determined prior to the measurements. Frequency sweeps in the melt were performed at 130 °C, 150 °C and 170 °C.

SUPPORTING INFORMATION

Statistical Analysis

To ensure the reproducibility of the reported results and to enable statistical analysis all batch experiments were conducted independently. For statistical analysis of density measurements mean values and standard deviations (table S10 and table S11) were examined using a sample size of five (n=5). For statistical analysis of tensile tests mean values and standard deviations (table S7-S9) were calculated using a sample size of six (n=6). Highest and lowest values were excluded. Origin Pro software was used to evaluate the reported data.

Synthetic Procedures

Synthesis of the titanium precatalyst **[Ti]**:
The synthesis of the titanium precatalyst [Ti] (N‘,N“-Bis(2,6-diisopropylphenyl)-N,N-diisopropylguanidinatotrichloridotitanium (IV)) was conducted based on published procedure by amine elimination reaction using diethylamidotrichloridotitanium(IV) instead of dimethylamidotrichloridotitanium (IV) as precursor[1,2].

Synthesis of the zirconium precatalyst **[Zr]**:

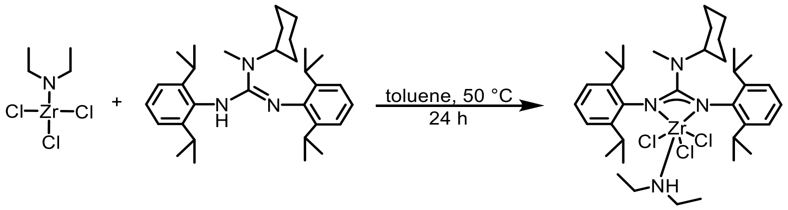

Scheme 1: Synthesis of the zirconium precatalyst **[Zr]**.

In a Schlenk flask, 1.5 g (3.15 mmol, 1.0 eq) 1-Cyclohexyl-2,3-bis(2,6-diisopropyl-phenyl)-1-methylguanidine [Gua^{CyMe}H] was dissolved in 25 mL dry toluene. 1.08 g (3.47 mmol, 1.1 eq) [$ZrCl_3NEt_2$]·Et_2O, synthesized according to published procedure[3], was added. The solution was stirred for 24 hours at 50 °C. The solution was cooled to room temperature and filtrated. The solvent was removed on reduced pressure. The residue was extracted with 5 mL toluene. After crystallization at -27 °C from hexane for 12 hours colorless crystals were yielded.
^{1}H-NMR (300 MHz, C_6D_6): δ = 7.20-7.04 (m, 6H), 4.07-4.04 (t, 4H), 3.51-3.45 (tt, 1H), 2.65 (*b*s, 4H), 2.04 (s, 3H), 1.66 (*b*d, 12H), 1.39-1.37 (d, 12H), 1.34-1.22 (m, 5H), 0.96-0.78 (m, 4H), 0.74-0.70 (t, 6H), 0.62-0.51 (m, 1H) ppm.

Polymer Synthesis
All terpolymerizations were conducted in a 300 mL glass autoclave (BüchiGlasUster) with a mechanical stirrer (500-1000 rpm). The temperature of the glass autoclave was controlled via an external water bath. The synthesis of all long chain alcohols (copolymerizations and oxidations) were conducted in a 1000 mL temperature and pressure controlled stainless steel autoclave with a mechanical stirrer (500-1000 rpm). All precatalysts, TEA, comonomers and activator were added as stock solutions in toluene. During the polymerization (semi batch mode) the ethylene pressure was kept constant by replenishing ethylene flow. The ethylene consumption was measured with a Bronkhorst High-Tech EI-Mass-Flow controller in liter normal (L_n), 1 mol gas = 22.41 L_n at p = 1.01325 bar and 0 °C. After the reaction 10 mL ethanol *p.a.* was added to stop the reaction.

General Synthesis of the unsaturated backbone **P1**:
For the **P1** synthesis the glass autoclave was evacuated (30 minutes) and heated to 30 °C. The autoclave was charged with 50 mL dry toluene, stirred at 1000 rpm and pressured with ethylene (2 bara). 20 mmol VNB, 100 mmol 1-hexene, 0.5 mmol TEA and 3 mmol *d*-MAO were subsequently added as stock solutions in toluene so that the polymerization was conducted in 75 mL toluene/comonomer-solution. 6 µmol precatalyst **[Ti]** were added to start the reaction. After 6 L_n ethylene consumption the polymerization was quenched with ethanol. The polymer solution was washed with H_2O and HCl, and the water-phase was extracted with toluene. The organic phases were combined, and all volatiles were removed under reduced pressure. The synthesis of **P1** was repeated in three independent batch experiments. The results are summarized in table S1.

Table S1: GPC data of the **P1** batch mode synthesis.

Batch	TEA [mmol]	V_{eth} [L_n]	M_n [g/mol]	Đ
**1**	0.5	6	16 500	6.0
**2**	0.5	6	14 900	3.1
**3**	0.5	6	13 200	3.2

General Synthesis of long chain alcohols:

The steel autoclave was evacuated (30 min) and heated to 70 °C. The autoclave was charged with 150 mL dry toluene, stirred at 1000 rpm, and pressured with ethylene (2 bara). TEA, 0.5 mmol *d*-MAO and 1 µmol precatalyst were subsequently added as stock solutions in toluene to reach total 175 mL solution. For the synthesis of **P3** also 100 mmol 1-hexene were added. After desired ethylene consumption ethylene was removed and pure oxygen (3 bara) was pressured to the autoclave and stirred at 70 °C and 1000 rpm for 3 hours. The reaction was quenched with ethanol. The polymer was precipitated from ethanol, washed with HCl/H_2O, filtrated, washed with acetone, and dried in the oven (70 °C). The synthesis of **P3** was repeated in two independent batch experiments where the amount of TEA and the conversion of ethylene was varied. The results are listed in Table S2.

Table S2: GPC data of the **P3** batch mode synthesis.

Batch	TEA [mmol]	V_{eth} [L_n]	$\bar{M}_n$ [g/mol]	Đ
**1**	2.5	8	1 370	1.6
**2**	5	16	1 350	1.4

The authors of reference 4 (supporting information) describe the length of the long chain branches per LDPE molecule, determined by AFM, in nm. A chain length of 10 nm corresponded to a molecular weight of around 900 g/mol. In addition, reference 5 (supporting information) gives the max. branch length as about 160 C atoms, which corresponds to a molecular weight of 2 240 g/mol, and more than 3 long chain branches per single LDPE molecule. Therefore the desired molecular weight of the branches (long chain alcohols) **P3** was chosen to around 1 400 g/mol to be within the range of the typical long chain branches of LDPE described in literature.[4,5]

Ethylene Flow

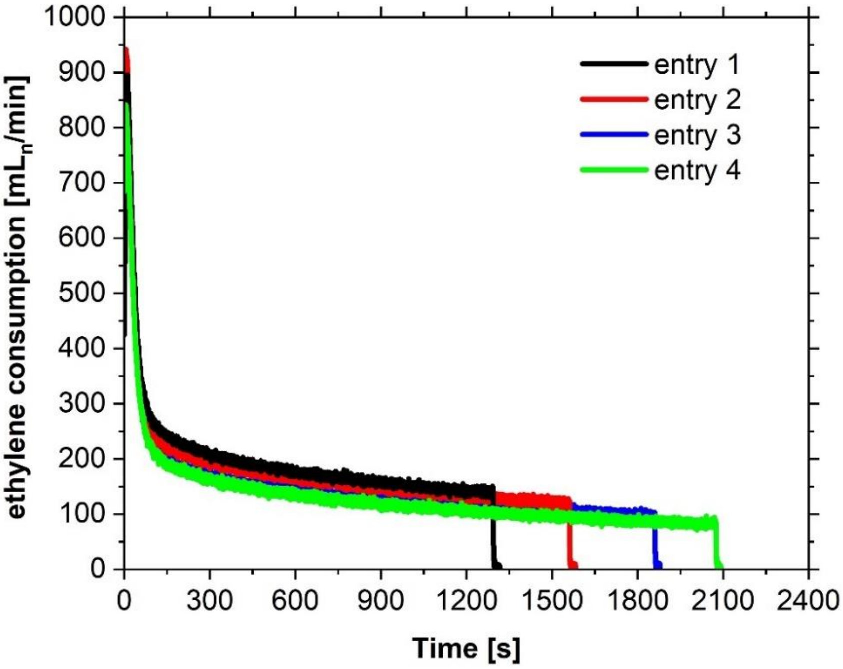

Figure S1: Ethylene flow for the ethylene/1-hexene/VNB terpolymerizations of Table 1 (entry 1-4).

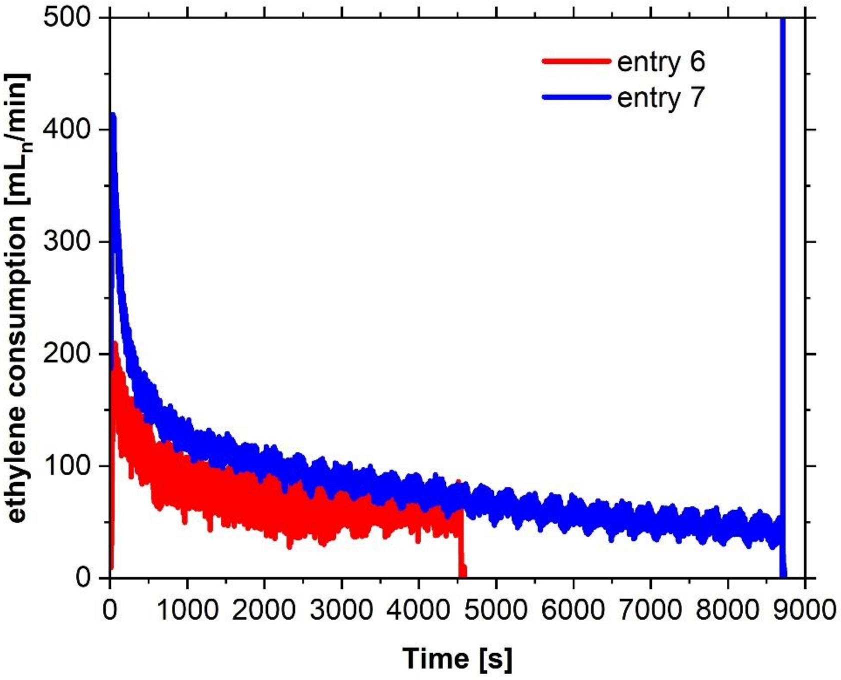

Figure S2: Ethylene flow of the ethylene/1-hexene copolymerizations of Table 2 (entry 6 and 7).

SUPPORTING INFORMATION

NMR Spectra

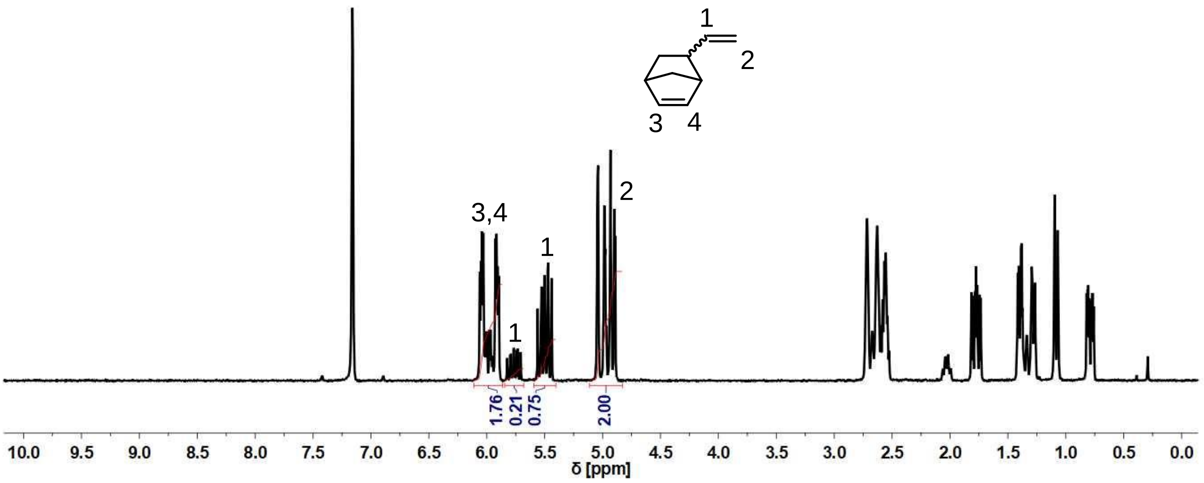

Figure S3: ^{1}H-NMR spectrum of vinylnorbornene (VNB) recorded in C_6D_6 at 25 °C.

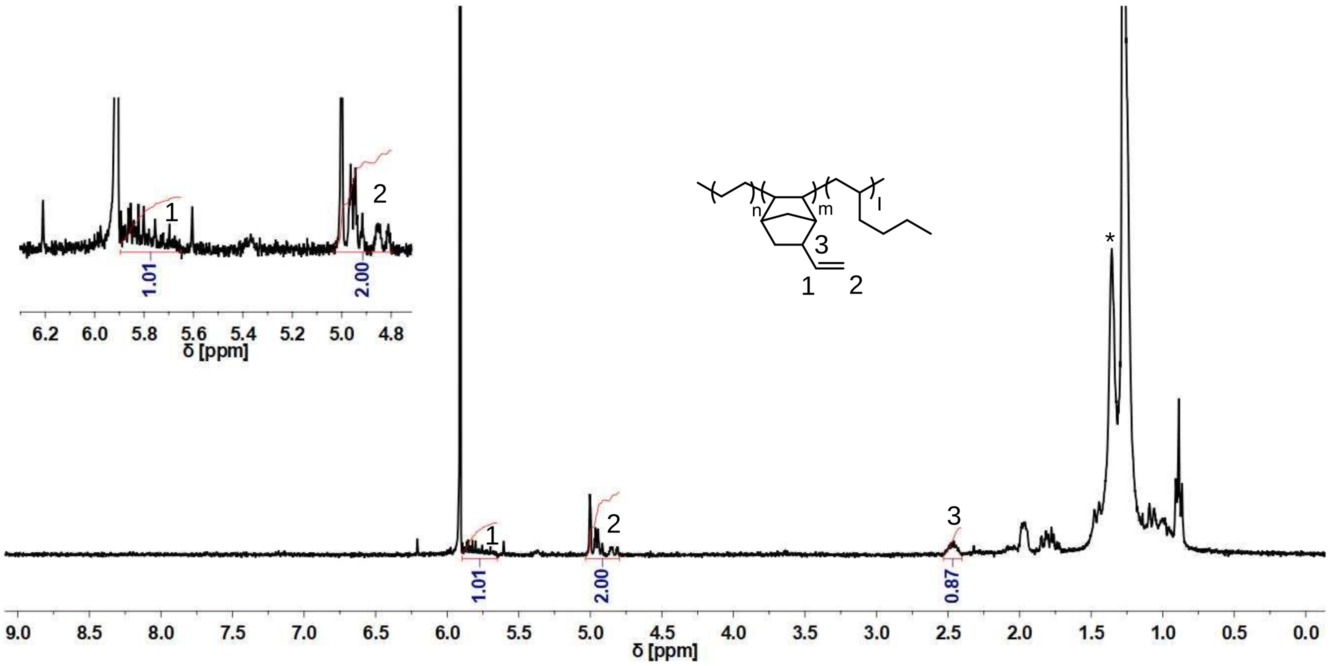

Figure S4: ^{1}H-NMR spectrum of ethylene/VNB/1-hexene terpolymer **P1** (Table S1, Batch 1) recorded in $C_2D_2Cl_4$ at 120 °C (*H_2O).

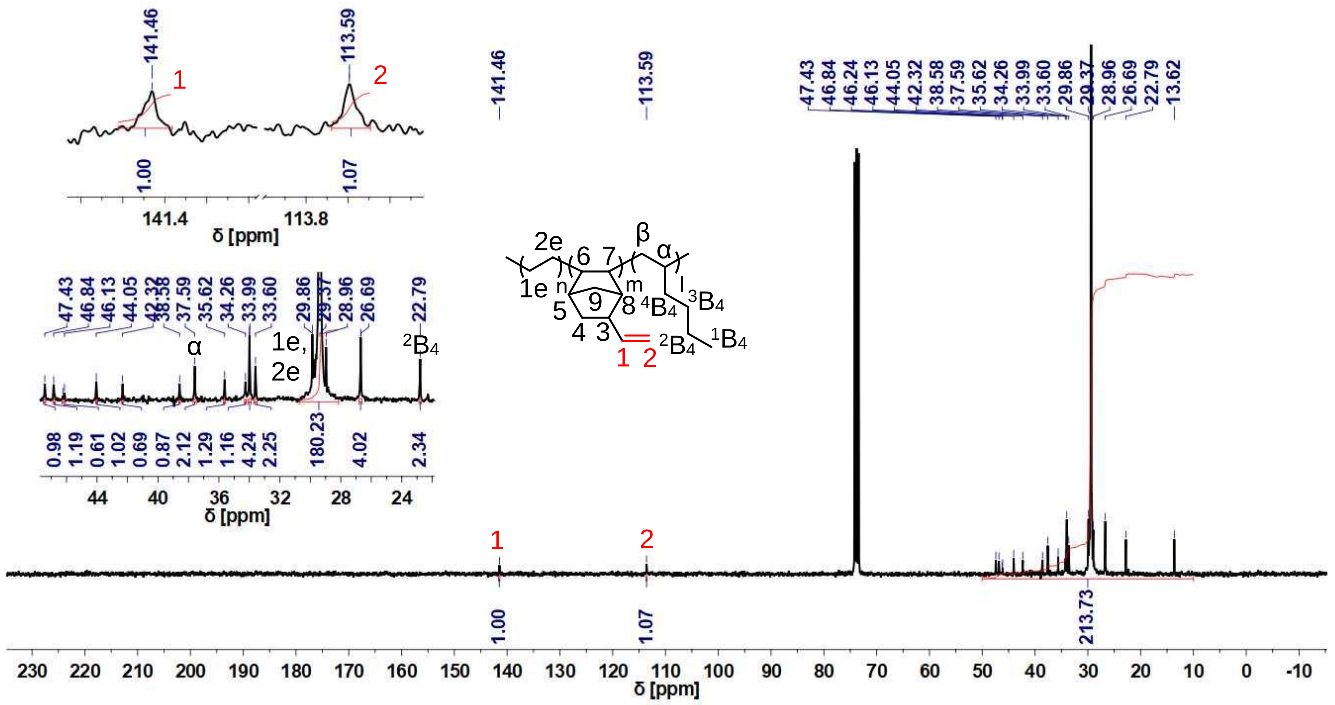

Figure S5: ^{13}C-NMR spectrum of ethylene/VNB/1-hexene terpolymer **P1** (Table S1, Batch 1) recorded in $C_2D_2Cl_4$ at 120 °C.

<u>Calculation of average vinylic- and 1-hexene-units per chain:</u>
As an example, the calculation of incorporated comonomer units per average polymer chain for the VNB/1-hexene/ethylene terpolymers is conducted for Table S1, Batch 1-3. For this purpose, the average polymer chain length Mn of the corresponding GPC data and the ^{13}C-NMR spectrum of the VNB/1-hexene/ethylene terpolymer (Figure S5) are used.

Table S3: ^{13}C-NMR data of the terpolymer **P1**.

δ [ppm]	assignment	name of the integral
141-142 + 113-114	(VNB C1 + VNB C2)·1/2	I_{VNB}
10-50	Ethylene C1 + Ethylene C2 + VNB C3-C9 + $^1B_4$-$^4B_4$ + α + β	I_{Total}
22-23 + 37-38	($^2B_4$ + α)·1/2	I_{Hex}

$$I_{Eth} = (I_{Total} - 7\cdot I_{VNB} - 6\cdot I_{Hex})\cdot 1/2$$

$$X_{VNB} = I_{VNB}/(I_{Eth}+I_{VNB}+I_{Hex}) \approx 1.1 \text{ mol\%}$$

$$X_{Eth} = I_{Eth}/(I_{Eth}+I_{VNB}+I_{Hex}) \approx 96.6 \text{ mol\%}$$

$$X_{Hex} = I_{Hex}/(I_{Eth}+I_{VNB}+I_{Hex}) \approx 2.3 \text{ mol\%}$$

To calculate the average vinylic- and 1-hexene-units per chain, the incorporation of the single monomers (mol %) and M_n is used. At first the average number of carbon atoms per chain N_C is determined.

$$N_C = M_n / 14 \text{ g/mol}$$

Next, the total number of carbons C_{total} is determined by using the calculated mol % for VNB, ethylene and 1-hexene units and their carbon count.

$$C_{total} = 9X_{VNB} + 6X_{Hex} + 2X_{Eth}$$

The average number of vinylic-units A_{Vin}, ethylene-units A_{Eth}, and 1-hexene-units A_{Hex} per chain is determined as follows:

$$A_{Vin} = X_{VNB} \cdot (N_c/C_{total})$$

$$A_{Hex} = X_{Hex} \cdot (N_c/C_{total})$$

$$A_{Eth} = X_{Eth} \cdot (N_c/C_{total})$$

Table S4: Calculation of average monomer units per polymer chain for **P1**.

Batch	$\bar{M}_n$ [g/mol]	Đ	A_{Vin}	A_{Hex}	A_{Eth}
**1**	16 500	6.0	6.0	12.5	525
**2**	14 900	3.1	5.4	11.3	474
**3**	13 200	3.2	4.8	10.0	420

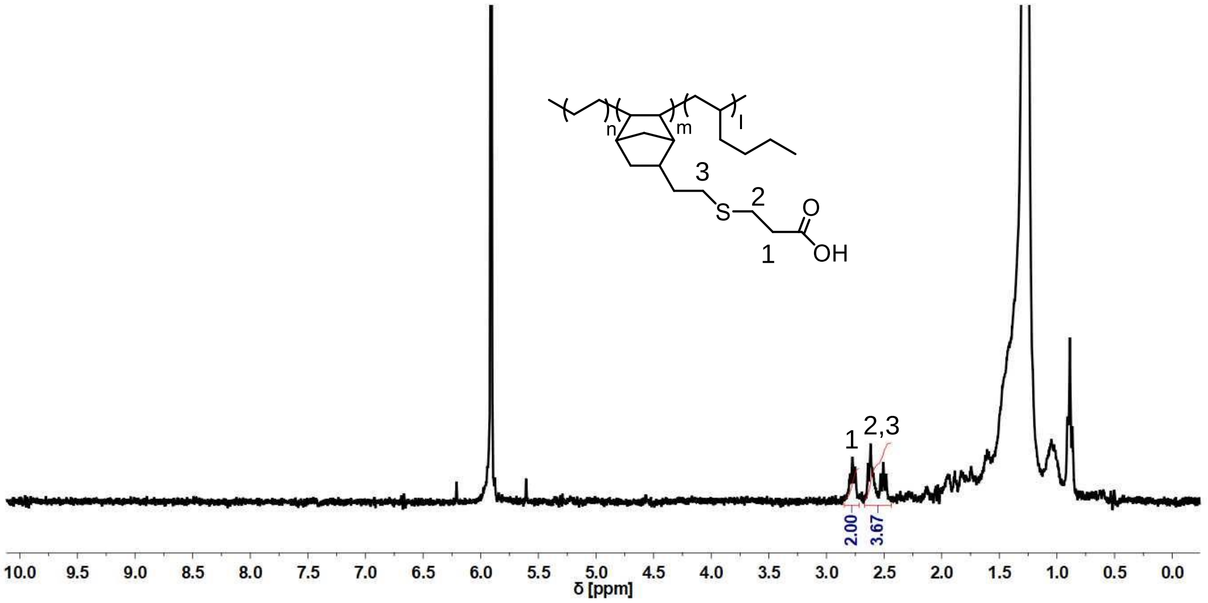

Figure S6: ^{1}H-NMR spectrum of the carboxylic acid functionalized terpolymer **P2** in $C_2D_2Cl_4$ at 120 °C.

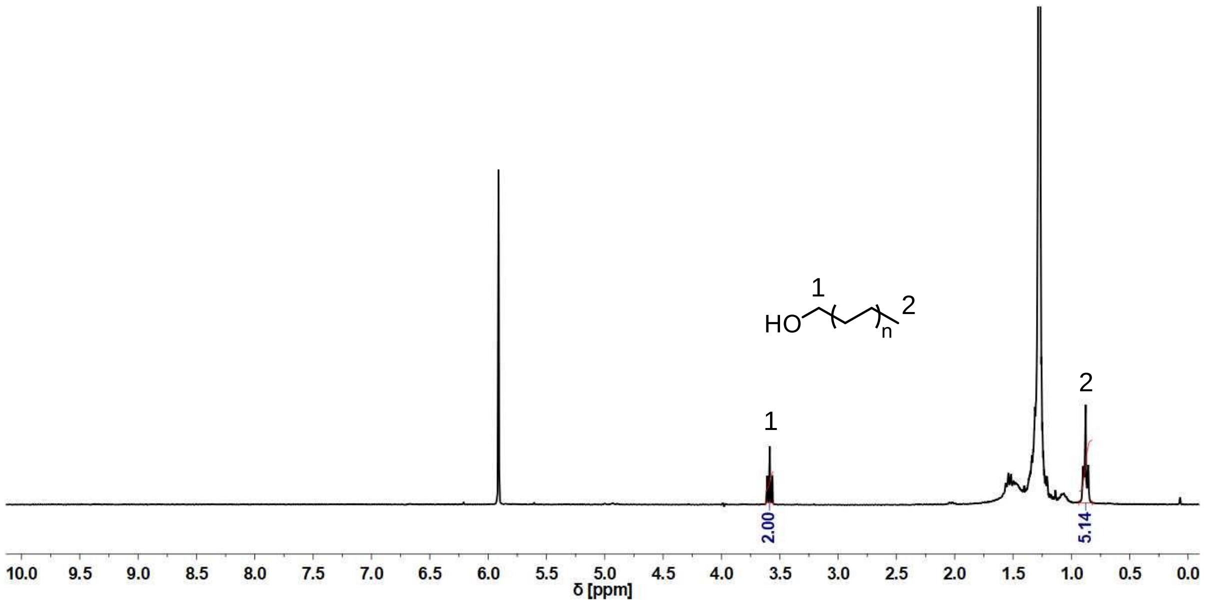

Figure S7: ^{1}H-NMR spectrum for the PE-OH of (Table 2, entry 5) in $C_2D_2Cl_4$ at 120 °C.

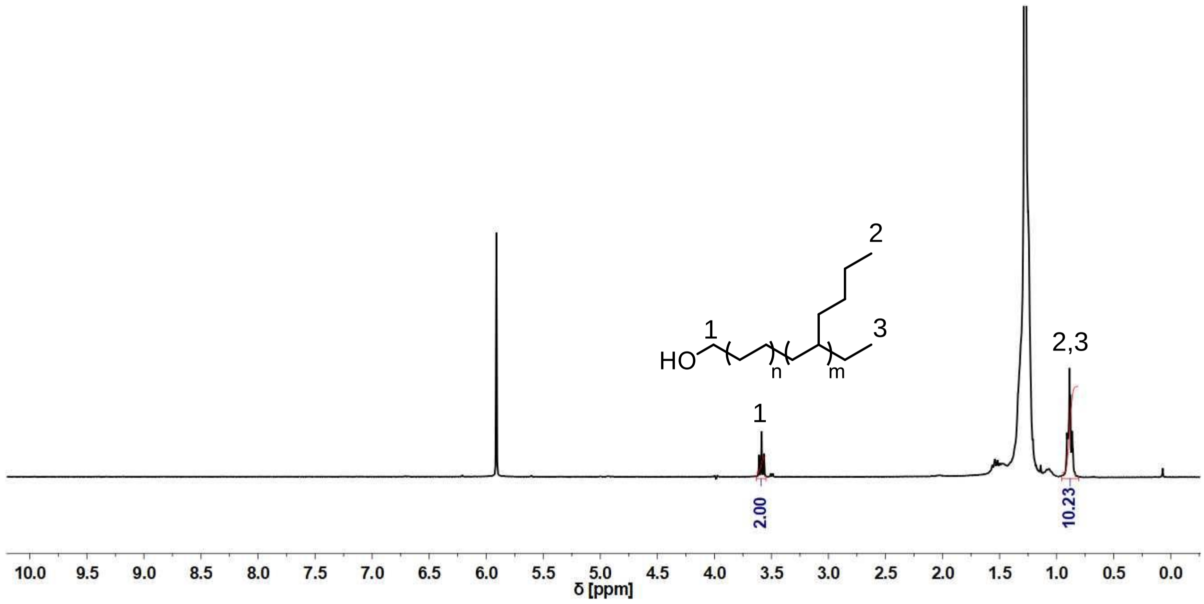

Figure S8: ^{1}H-NMR spectrum of **P3** (Table S2, Batch 1) in $C_2D_2Cl_4$ at 120 °C.

Calculation of the degree of hydroxy functionalization:

To determine the degree of hydroxy functionalization for the desired chain length of the ethylene (1-hexene) (co)polymers the 1H-NMR spectrum for the PE-OH homopolymer (Figure S7) was used. Therefore, the resonances of the protons next to the hydroxy functionalization CH_2-OH 1 were set to 2.00. The degree of functionalization **x** was determined as follows:

$$I_{Res(CH3)} = I_2 - 3.00$$

$$I_{Res(norm)} = I_{Res(CH3)} / 6$$

$$\mathbf{X} = 1 / (1+I_{Res(norm)})$$

$$\mathbf{X} = 1 / (1+0.36) = 0.735 \approx 74\ \%$$

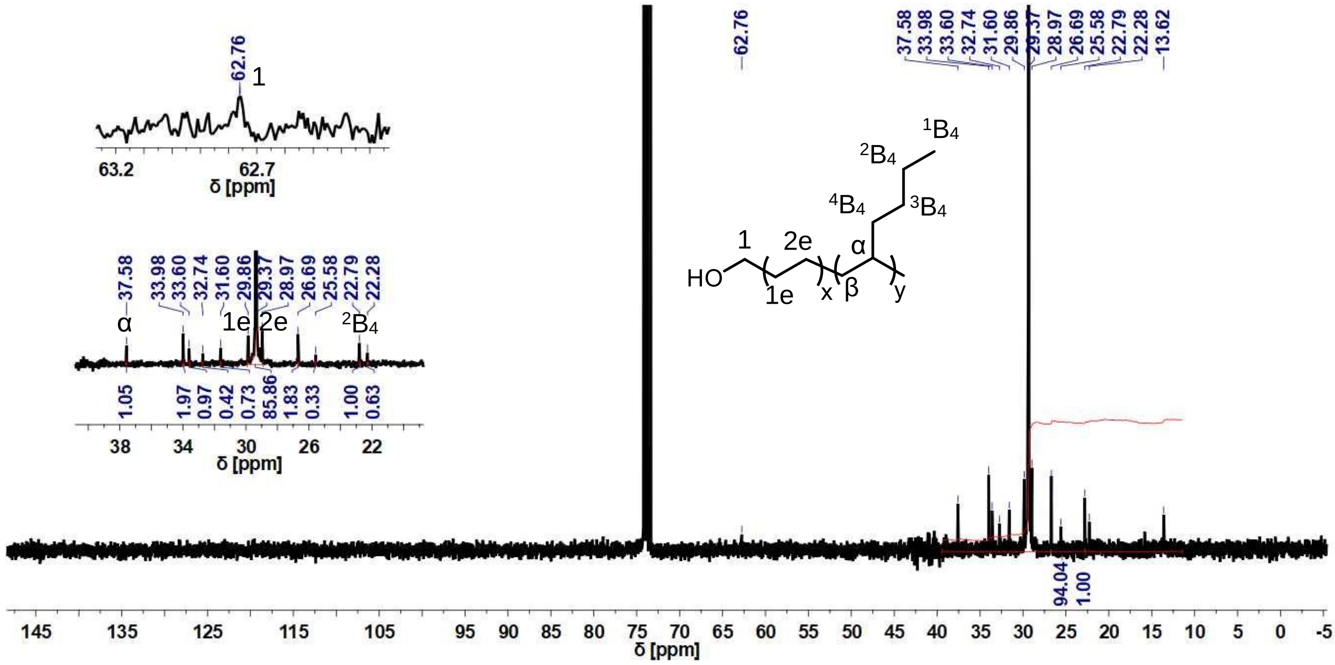

Figure S9: ^{13}C-NMR spectrum of **P3** (Table S2, Batch 1) in $C_2D_2Cl_4$ at 120 °C.

Calculation of incorporated 1-hexene-units per chain:

As an example, the calculation of incorporated 1-hexene units per average polymer chain is conducted for Table S2, Batch 1-2. For this purpose, the average polymer chain length M_n of the corresponding GPC data and the ^{13}C-NMR spectrum of the 1-hexene/ethylene copolymer (Figure S9) are used.

Table S5: 13C-NMR data of the terpolymer **P3**.

δ [ppm]	assignment	name of the integral
10-40	Ethylene C1 +Ethylene C2 + $^1B_4$-$^4B_4$ + α + β	I_{Total}
22.5-23 + 37-38	($^2B_4$ + α)·1/2	I_{Hex}

$$I_{Eth} = (I_{Total} - 6 \cdot I_{Hex}) \cdot 1/2$$

$$X_{Eth} = I_{Eth} / (I_{Eth} + I_{Hex}) \approx 97.7 \text{ mol\%}$$

$$X_{Hex} = I_{Hex} / (I_{Eth} + I_{Hex}) \approx 2.3 \text{ mol\%}$$

To calculate the average 1-hexene units per chain, the incorporation of the single monomers (mol %) and M_n is used. At first the average number of carbon atoms per chain NC is determined.

$$N_C = M_n / 14 \text{ g/mol}$$

Next, the total number of carbons C_{total} is determined by using the calculated mol% for ethylene and 1-hexene units and their carbon count.

$$C_{total} = 6X_{Hex} + 2X_{Eth}$$

The average number of ethylene-units A_{Eth}, and hexene-units A_{Hex} per chain is determined as follows:

$$A_{Hex} = X_{Hex} \cdot (N_C/C_{total})$$

$$A_{Eth} = X_{Eth} \cdot (N_C/C_{total})$$

Table S6: Calculation of average monomer units per polymer chain for **P3**.

**Batch**	**$\bar{M}_n$ [g/mol]**	**Đ**	**A_{Hex}**	**A_{Eth}**
**1**	1 370	1.6	1.0	46
**2**	1 350	1.4	1.0	45

Polymer Functionalization

General synthesis of the acid-functionalized backbone ***P2****:*

In a Schlenk flask, 3.50 g **P1** (Batch 1, Table S1, ~4.3 wt.-% VNB, 1.26 mmol olefin units 1.0 eq), 1.34 g (12.6 mmol, 10 eq) 3-mercaptopropionic acid and 103 mg (0.63 mmol, 0.5 eq) AIBN were dissolved in 120 mL dry toluene and heated under an argon atmosphere to 75 °C. The reaction was stirred for 3-4 hours. The polymer was precipitated by adding ethanol, filtrated, washed with acetone, and dried in the oven (70 °C) under reduced pressure.

General synthesis of the LDPE mimic ***P4****:*

In a Schlenk flask, 0.50 g **P2** (M_n = 18 000 g/mol, ~0.18 mmol carboxylic units, 1.0 eq), 0.29 g **P3** (Batch 2, Table S2, ~74 % hydroxy-functionalization, M_n = 1 350 g/mol, 0.22 mmol, 1.2 eq) and 60 mg *p*-TosOH were dissolved in 20 mL dry toluene and heated under an argon atmosphere to 130 °C. The solution was stirred under constant argon flow for 18 hours. The polymer was precipitated by adding ethanol, filtrated, washed with acetone, and dried in the oven (70 °C) under reduced pressure.

General depolymerization of the LDPE mimic ***P4****:*

In a pressure tube 500 mg LDPE-mimic **P4** and 50 mg (0.15 mmol) tetra-*n*-butyl-ammonium bromide were suspended in a mixture of 1 mL NaOH (1 N) and 9 mL toluene. The mixture was heated under argon to 110 °C for 18 h. Afterwards, 10 mL toluene were added for dilution. The polymer was precipitated by adding ethanol, filtrated, washed with acetone, and dried in the oven (70 °C) under reduced pressure.

General repolymerization of the LDPE mimic ***P4****:*

In a Schlenk flask, 400 mg of the depolymerized **P4**, and 60 mg p-TosOH were dissolved in 20 mL dry toluene and heated under an argon atmosphere to 130 °C. The solution was stirred under constant argon flow for 18 hours. The polymer was precipitated by adding ethanol, filtrated, washed with acetone, and dried in the oven (70 °C) under reduced pressure.

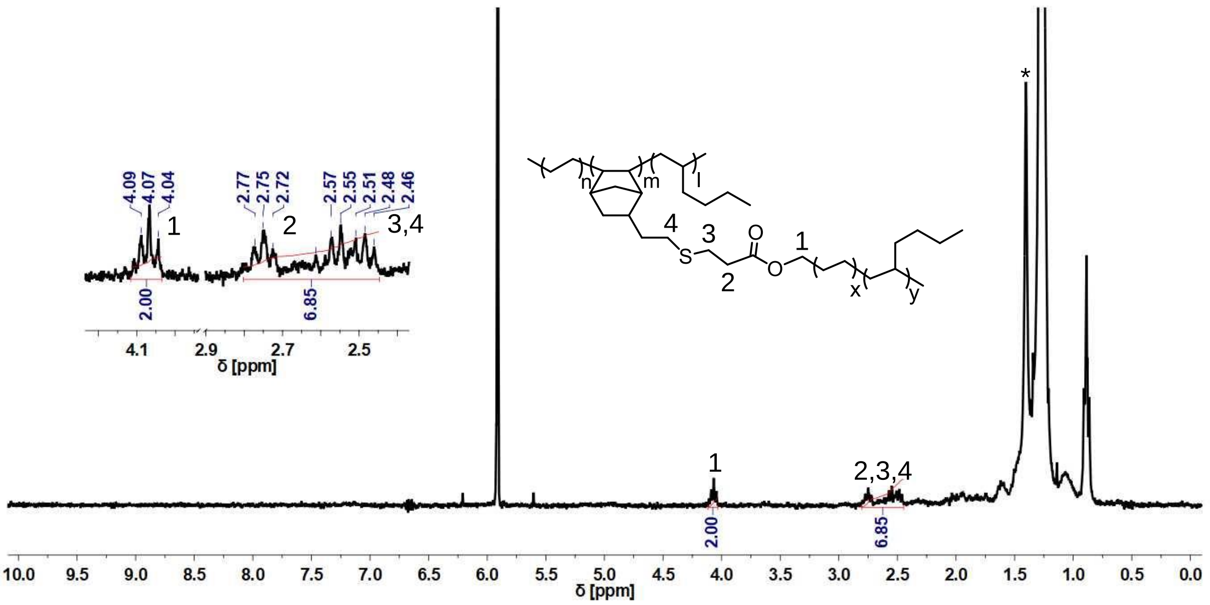

Figure S10: ^{1}H-NMR spectrum of the LDPE mimic **P4** in $C_2D_2Cl_4$ at 120 °C (*H2O).

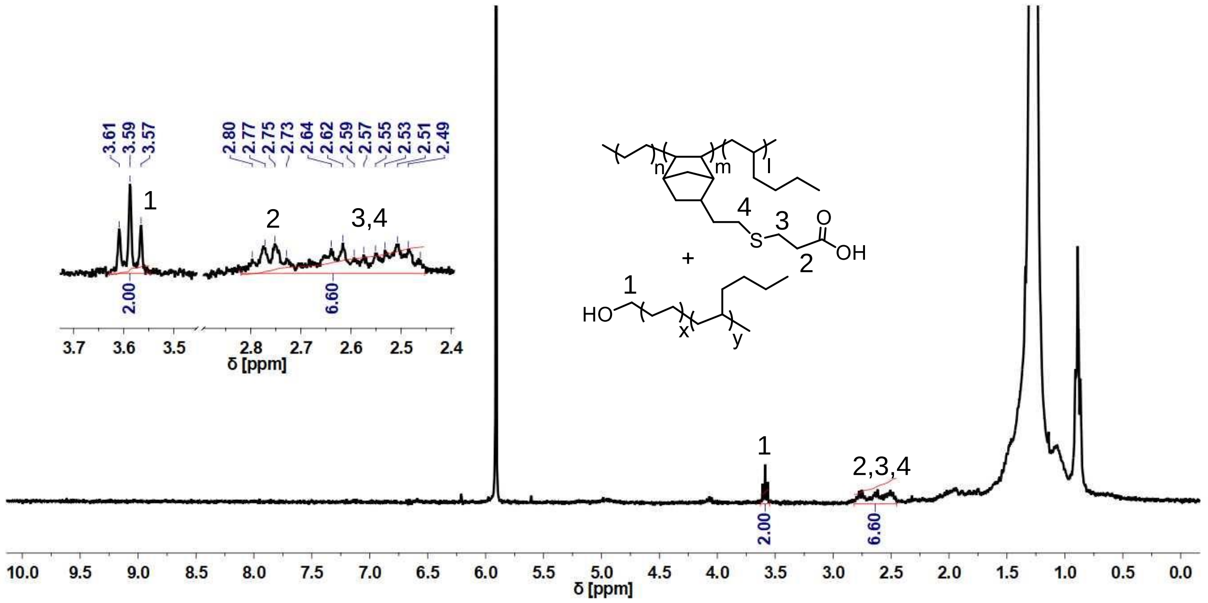

Figure S11: ^{1}H-NMR spectrum of the degrafted LDPE mimic **P4** in $C_2D_2Cl_4$ at 120 °C.

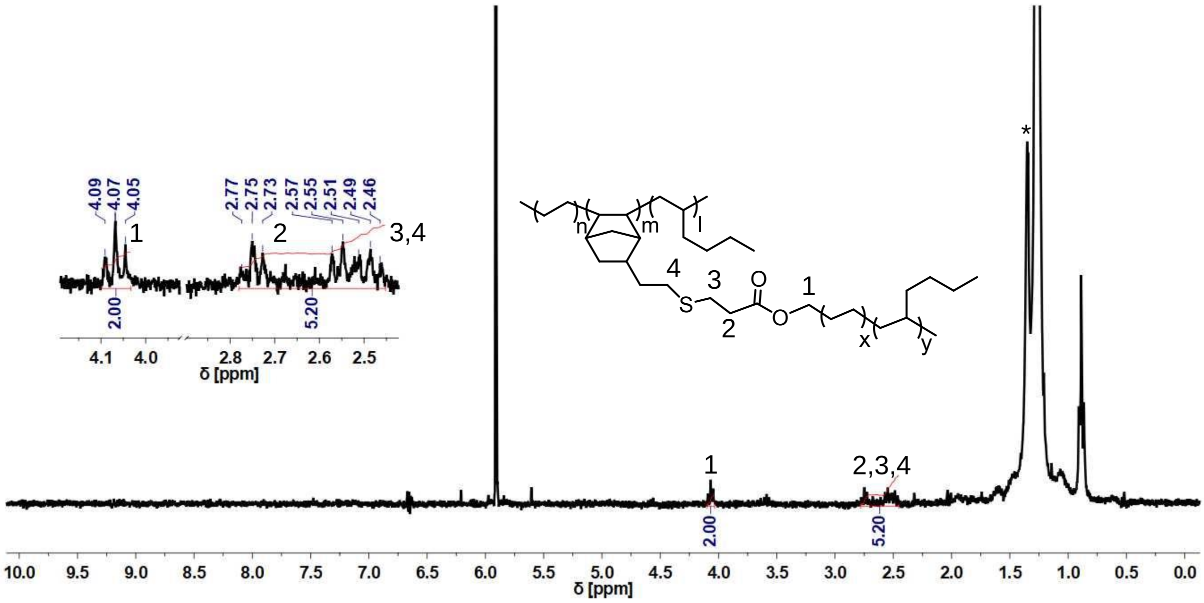

Figure S12: ^{1}H-NMR spectrum of the regrafted LDPE mimic **P4** (second grafting) in $C_2D_2Cl_4$ at 120 °C (**H*2O).

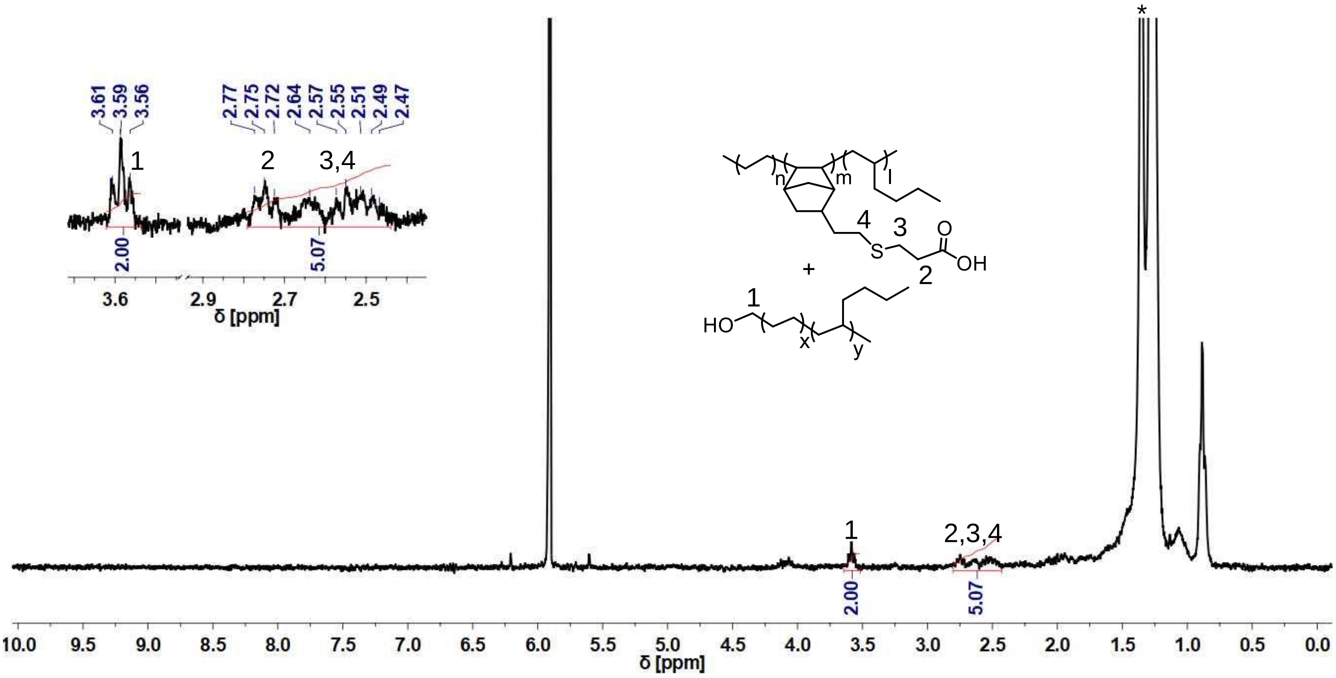

Figure S13: ^{1}H-NMR spectrum of the degrafted LDPE mimic **P4** (second degrafting) in $C_2D_2Cl_4$ at 120 °C (**H_2*O).

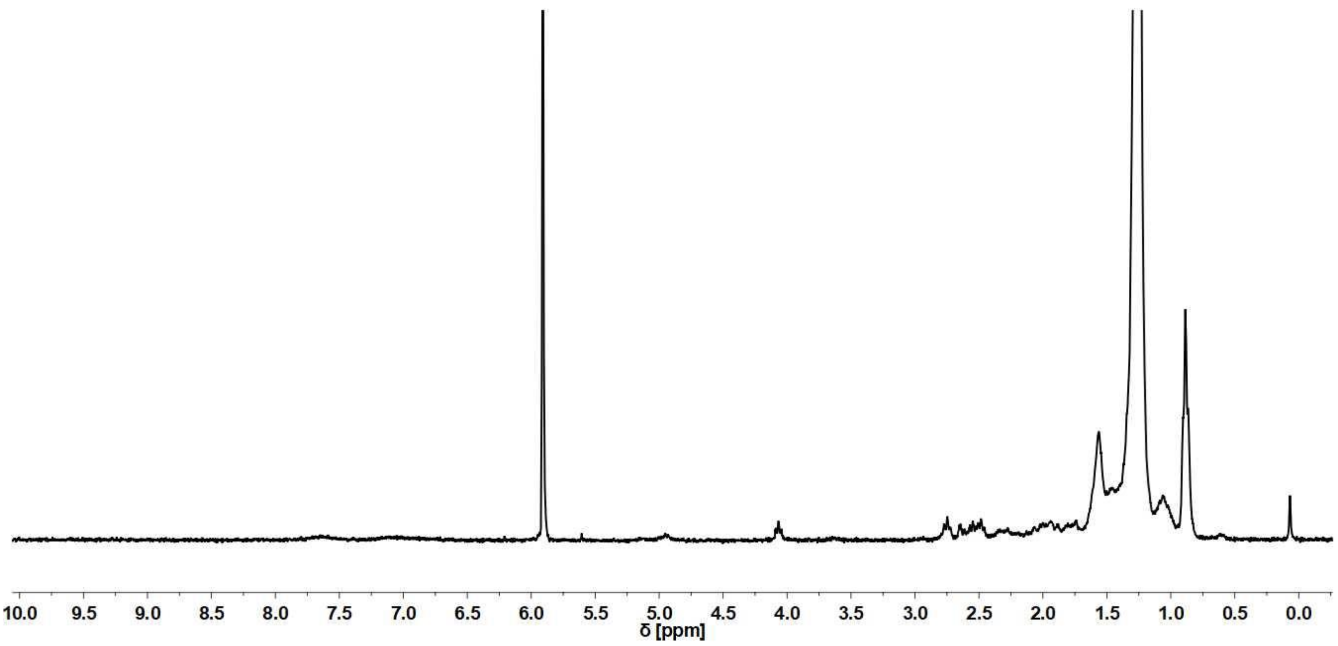

Figure S14: ^{1}H-NMR spectrum of the pure LDPE mimic **P4** batch used for rheology experiments before the addition of *Irganox 1010* in $C_2D_2Cl_4$ at 120 °C.

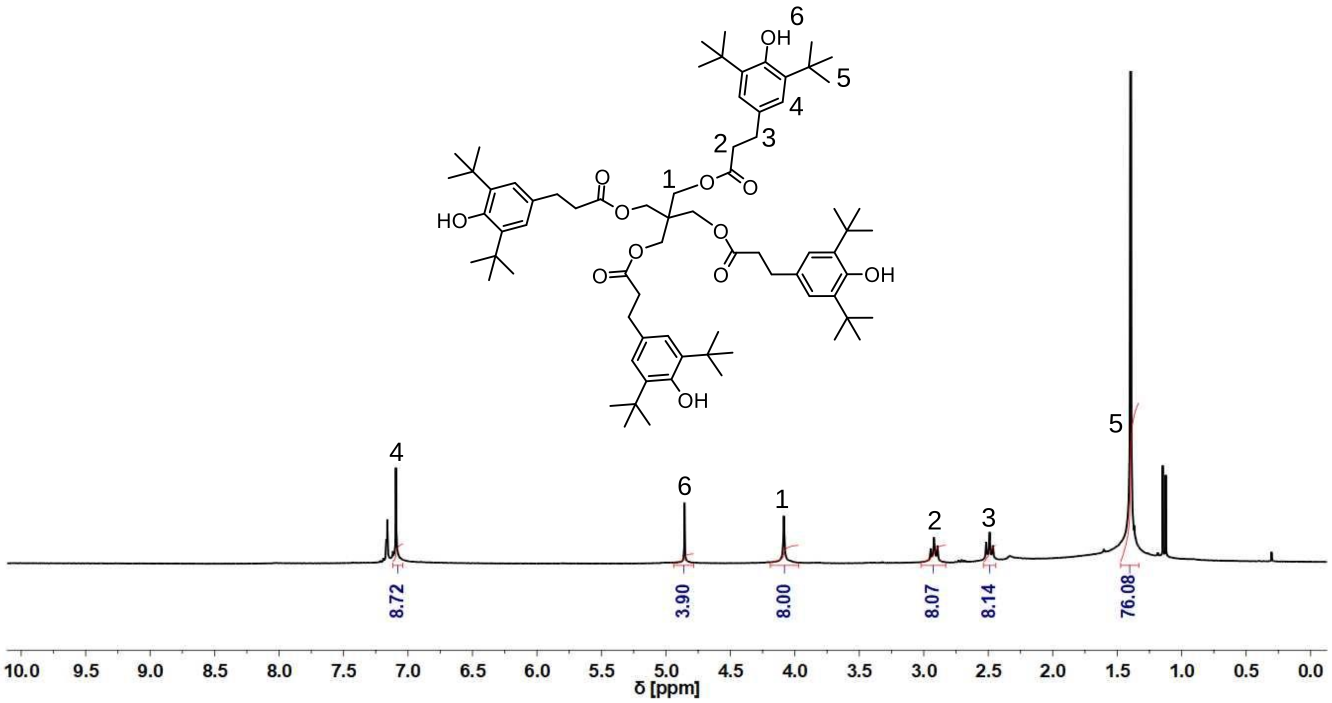

Figure S15: ^{1}H-NMR spectrum of the used stabilizer *Irganox 1010* in C_6D_6 recorded at 25 °C.

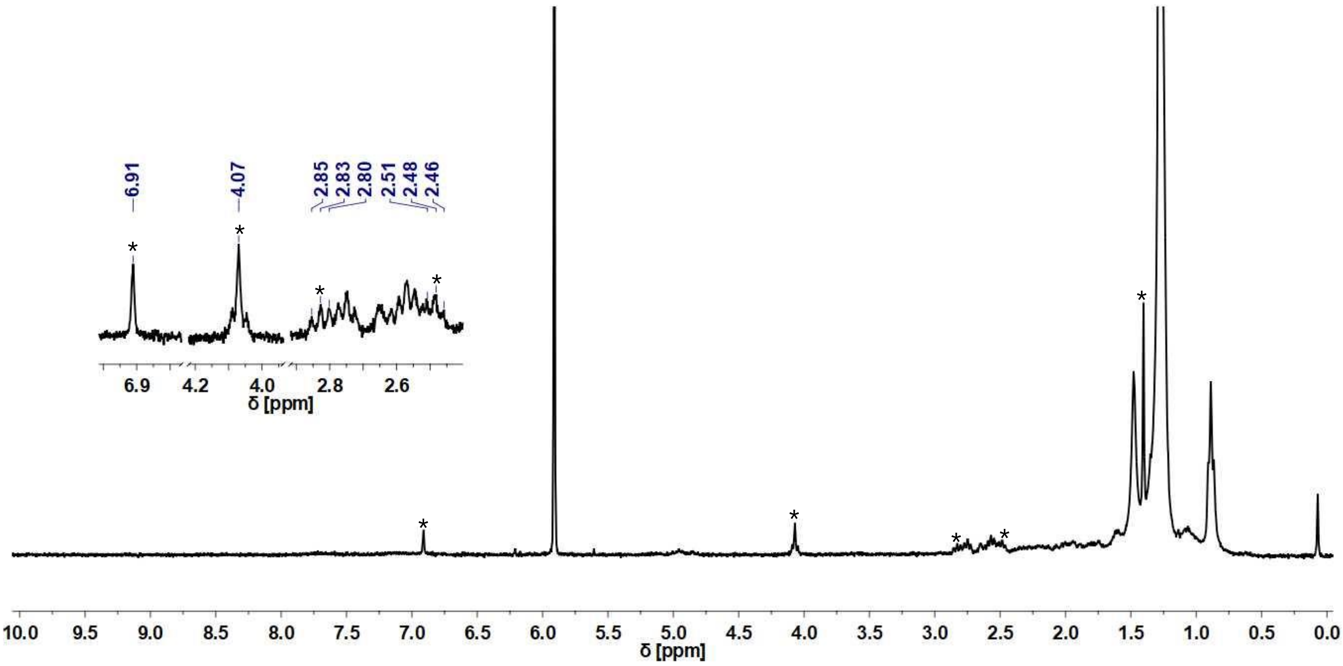

Figure S16: ^{1}H-NMR spectrum of the LDPE mimic **P4** (+ **Irganox 1010*) used for rheology experiments in $C_2D_2Cl_4$ at 120 °C.

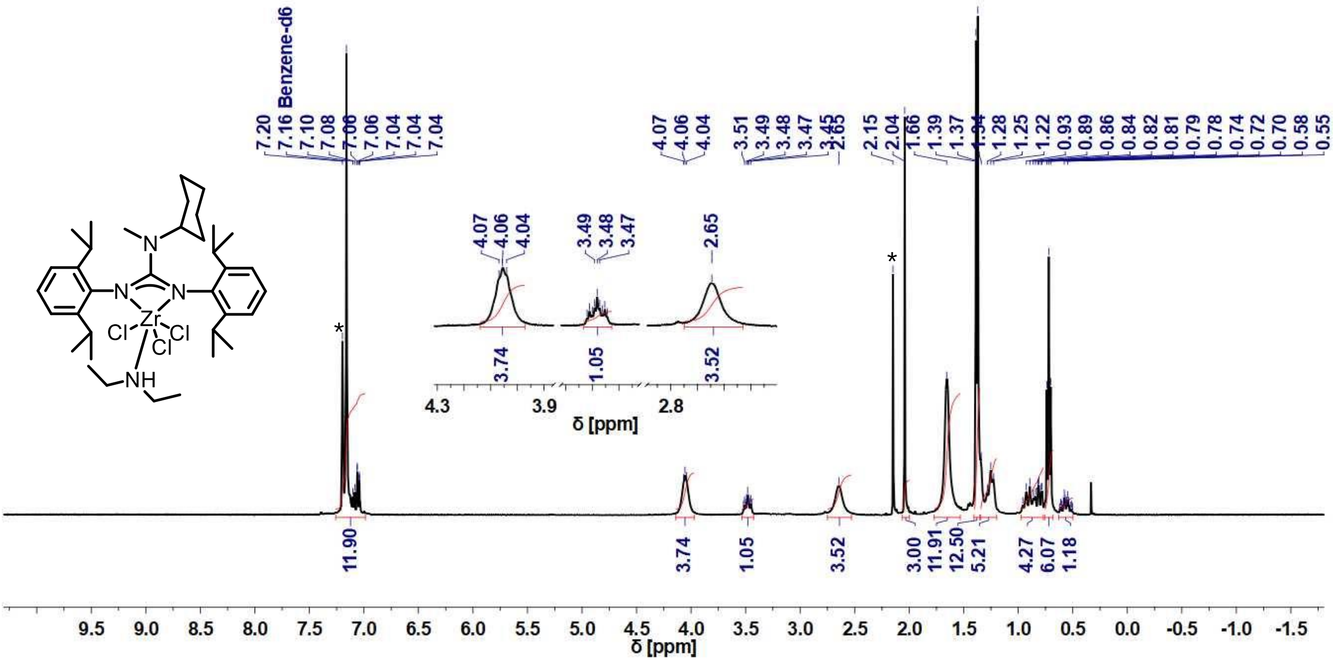

Figure S17: ^{1}H-NMR spectrum of the zirconium precatalyst **[Zr]** recorded in C_6D_6 (*toluene*).

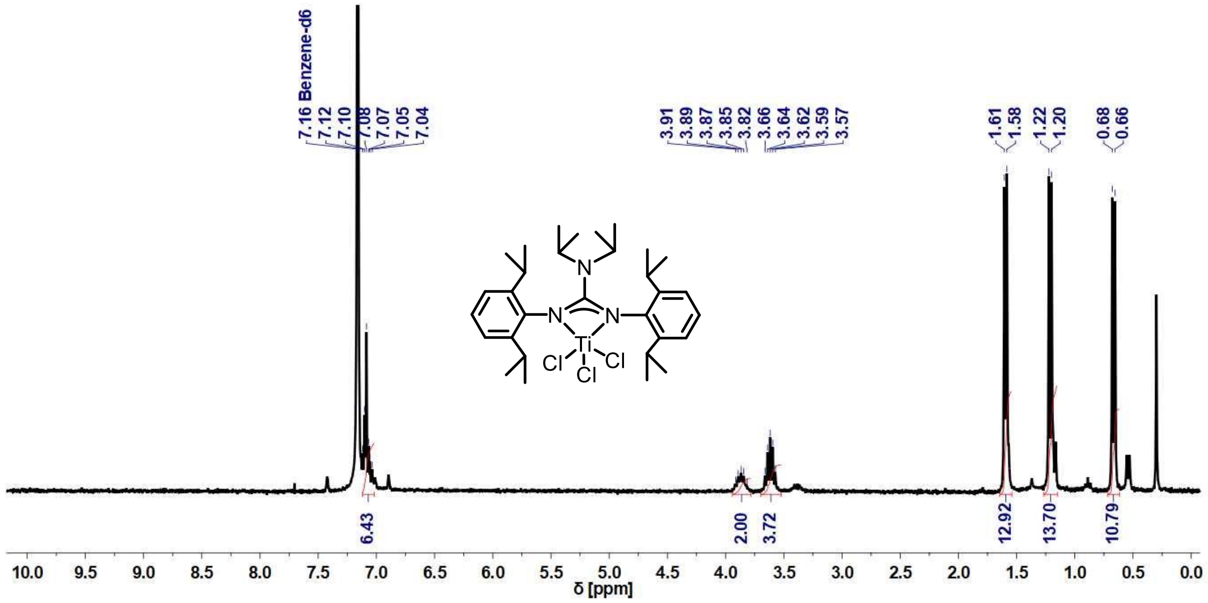

Figure S18: ^{1}H-NMR spectrum of the titanium precatalyst **[Ti]** recorded in C_6D_6.

GPC

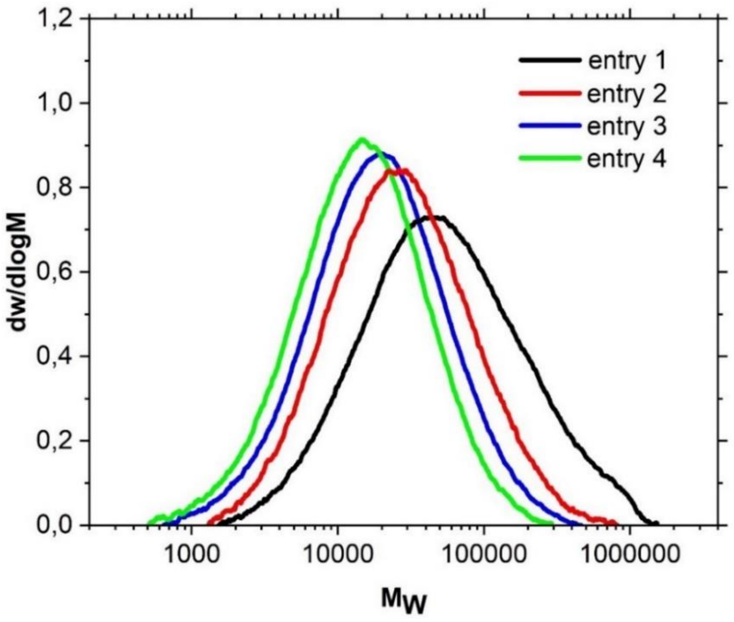

Figure S19: Molecular weight distributions of the ethylene/1-hexene/VNB terpolymers of Table 1 (entry 1-4).

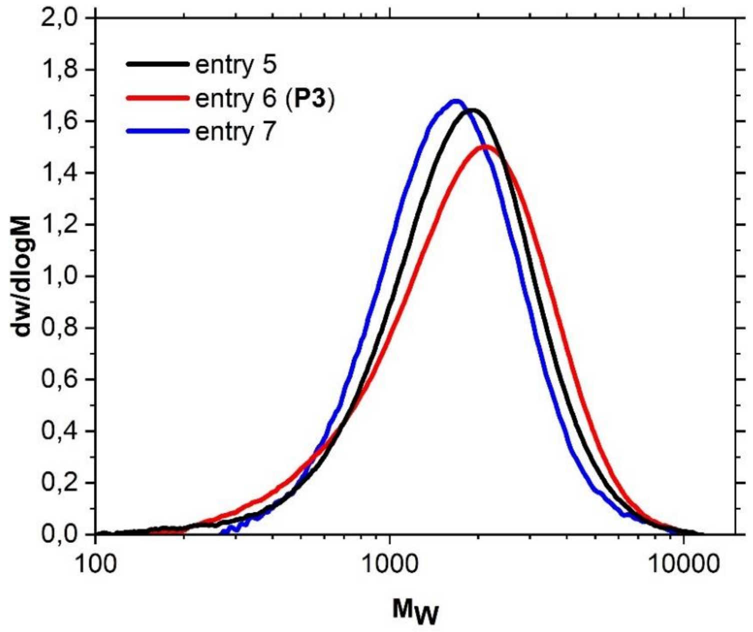

Figure S20: Molecular weight distributions of the ethylene/(1-hexene) (co)polymers of Table 2 (entry 5-7).

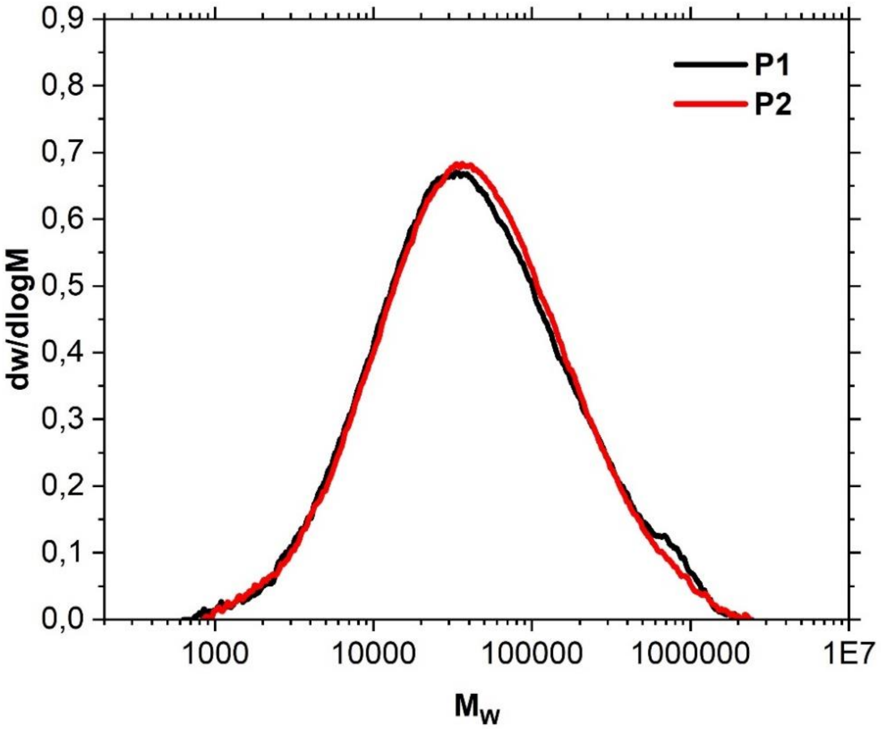

Figure S21: Molecular weight distributions of **P1** in comparison to **P2**.

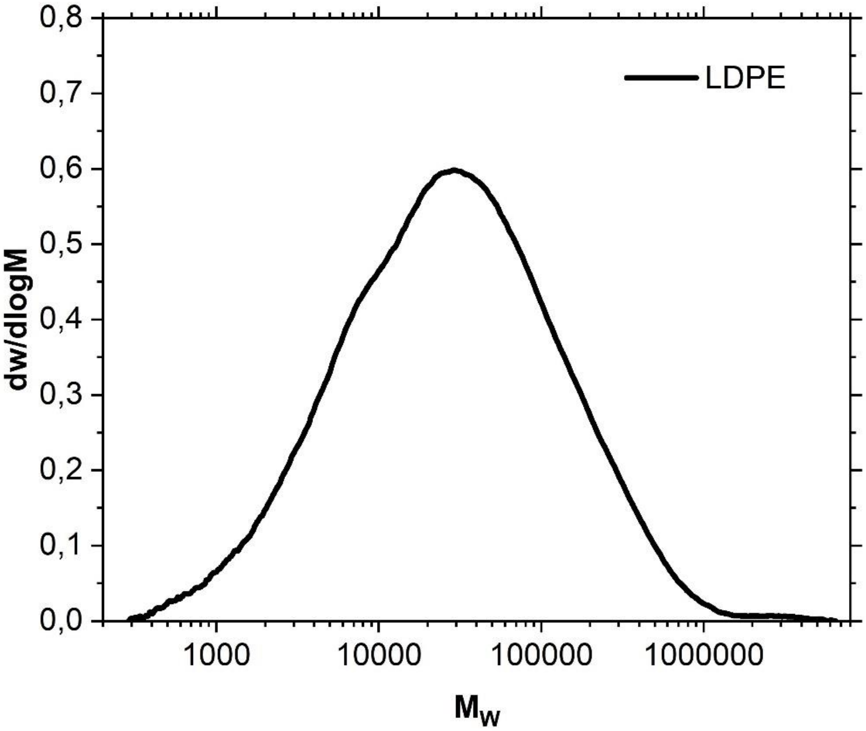

Figure S22: Molecular weight distributions of commercial LDPE Lupolen 1800P.

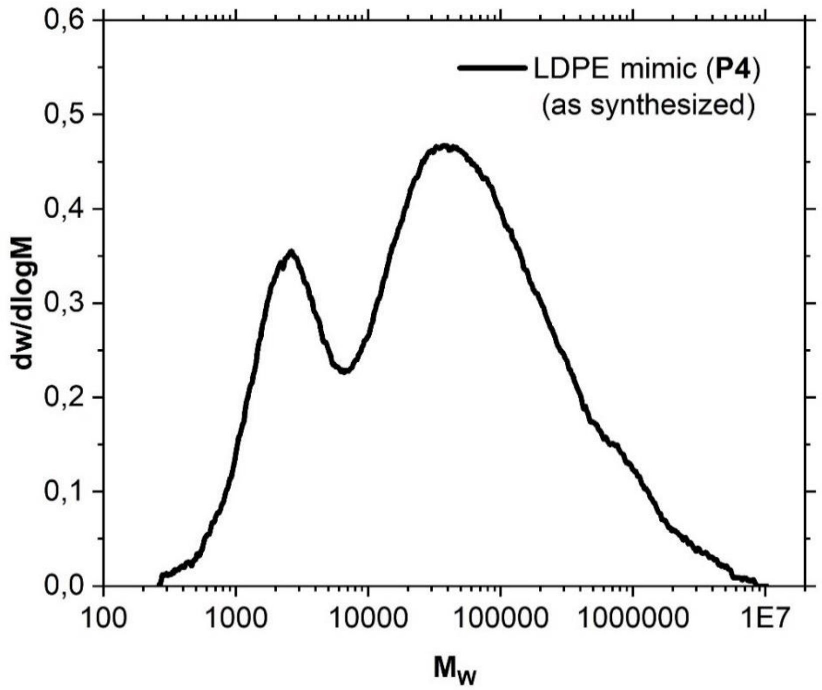

Figure S23: Molecular weight distributions of LDPE mimic **P4** (as synthesized).

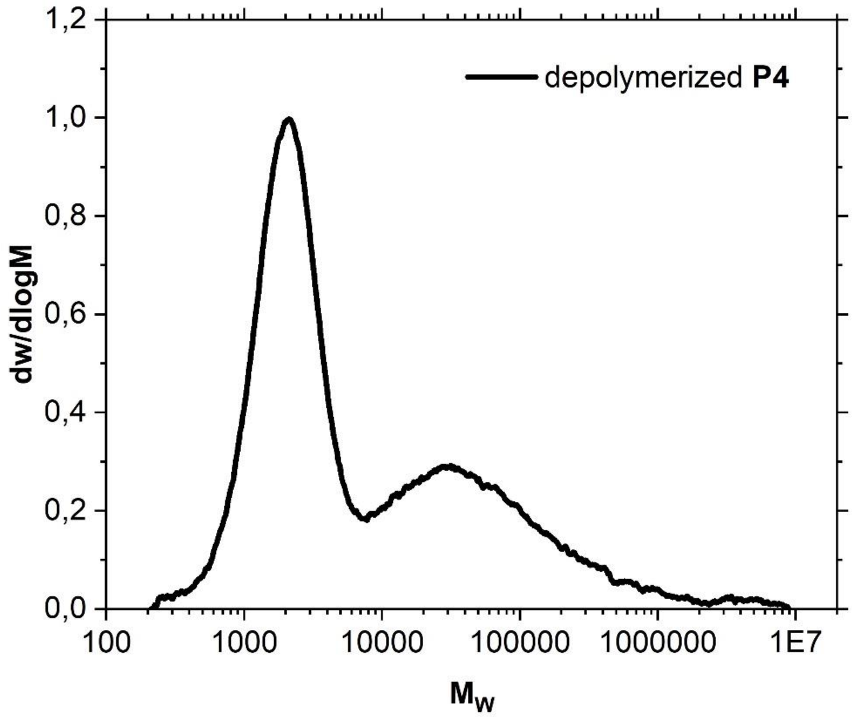

Figure S24: Molecular weight distributions of the degrafted LDPE mimic **P4**.

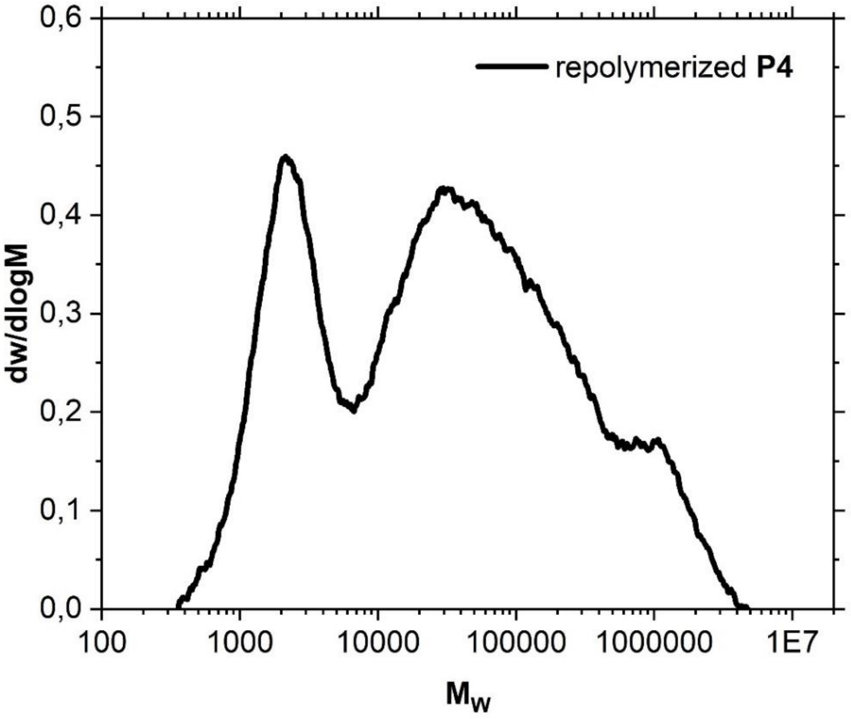

Figure S25: Molecular weight distributions of the repolymerized LDPE mimic **P4**.

DSC

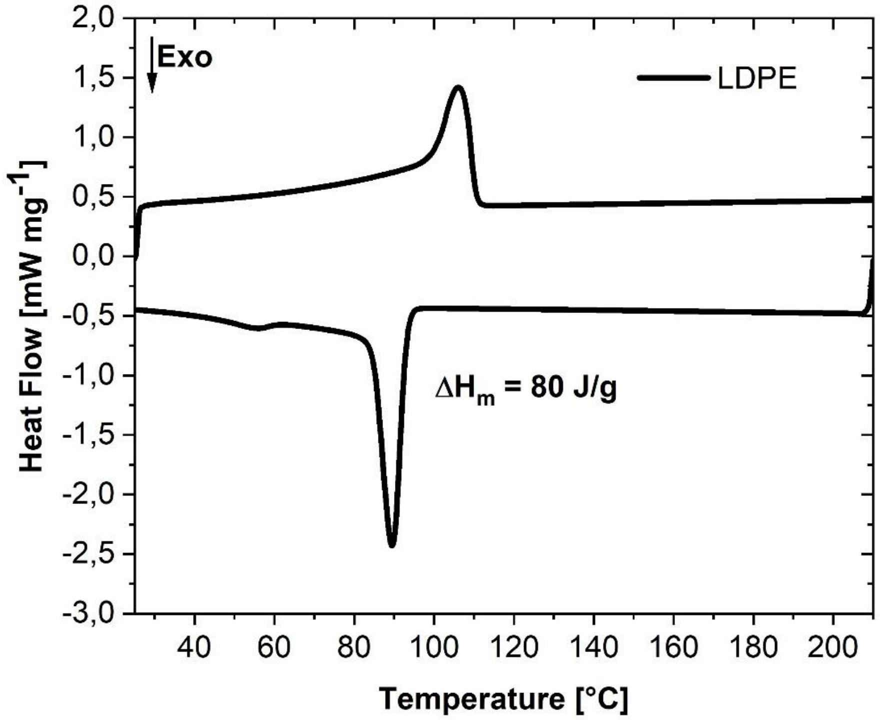

Figure S26: DSC (second heating curve) of the commercial LDPE Lupolen 1800P.

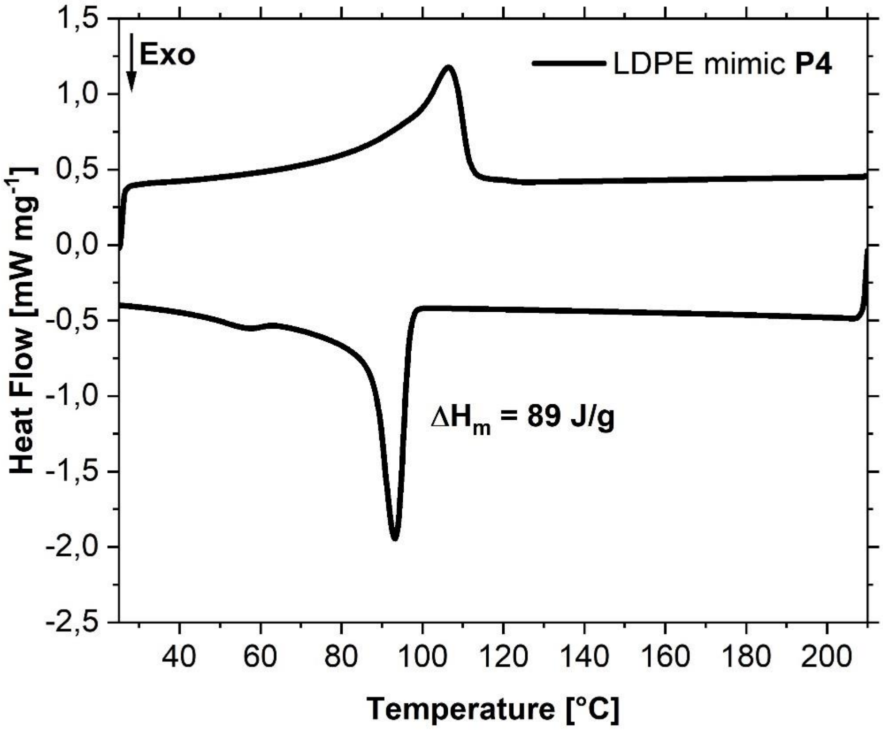

Figure S27: DSC (second heating curve) of LDPE mimic **P4.**

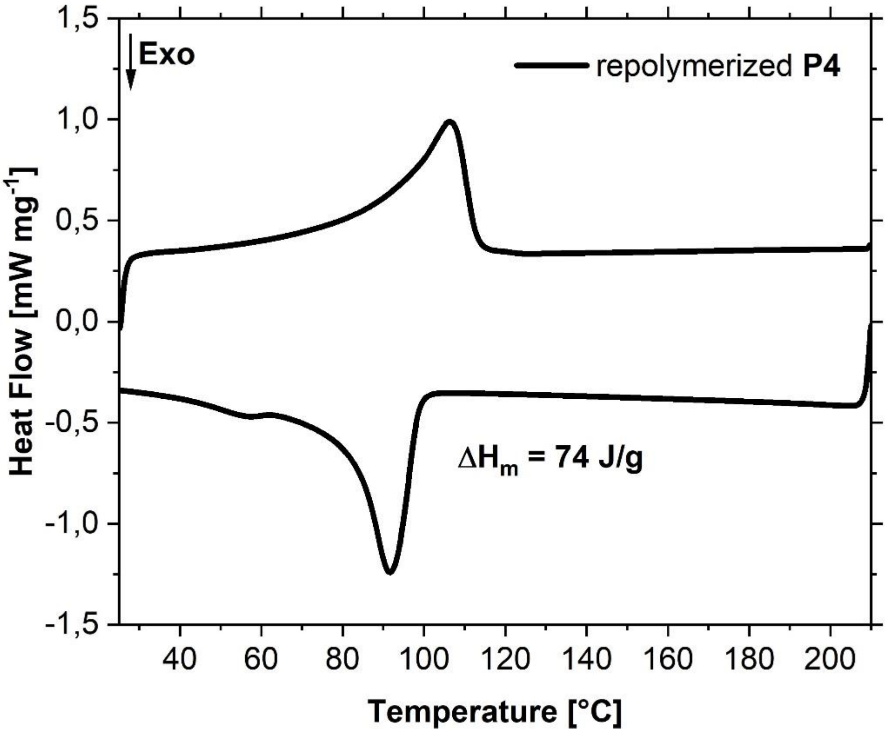

Figure S28: DSC (second heating curve) of recycled LDPE mimic **P4.**

TGA

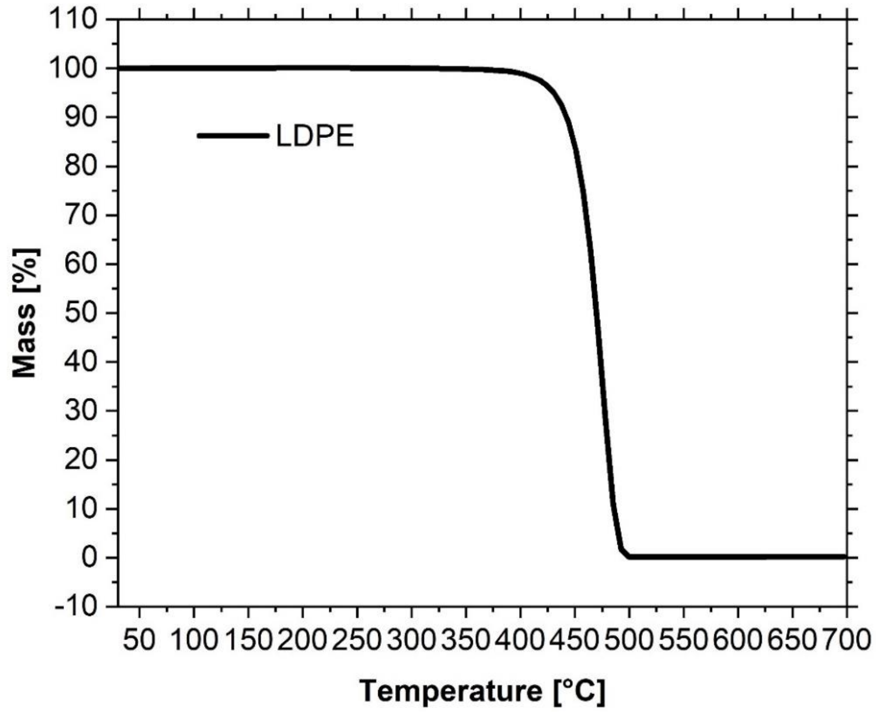

Figure S29: TGA analysis of Lupolen LDPE 1800P.

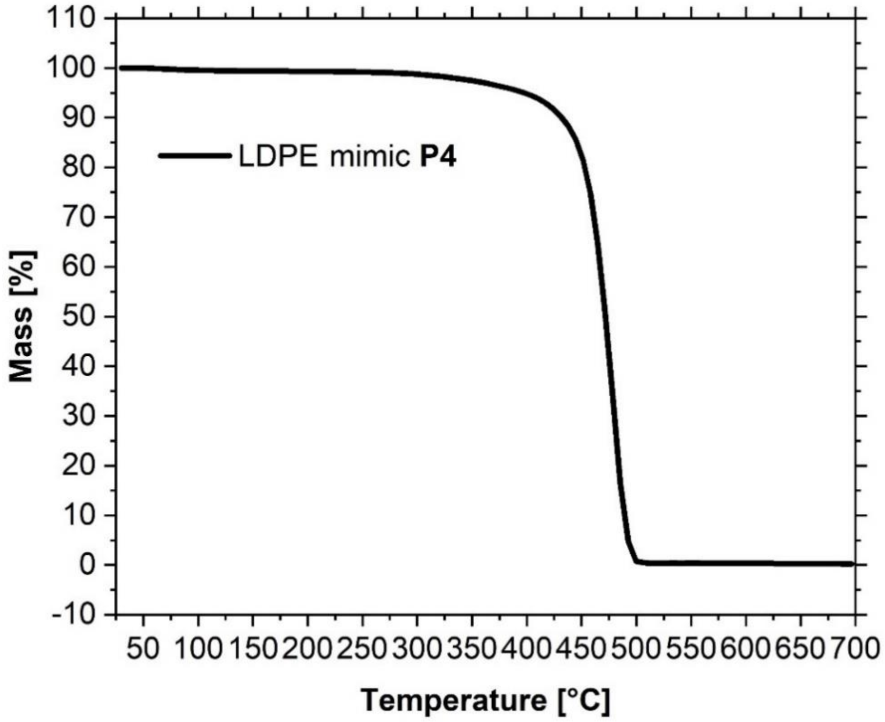

Figure S30: TGA analysis of LDPE mimic **P4**.

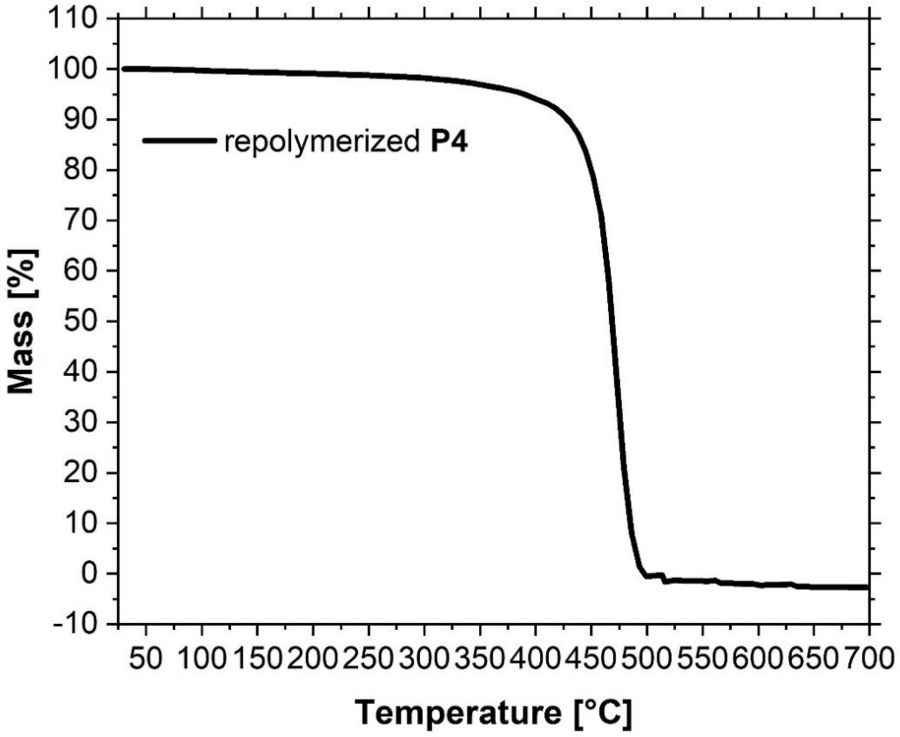

Figure S31: TGA analysis of recycled LDPE mimic **P4**.

Tensile Tests

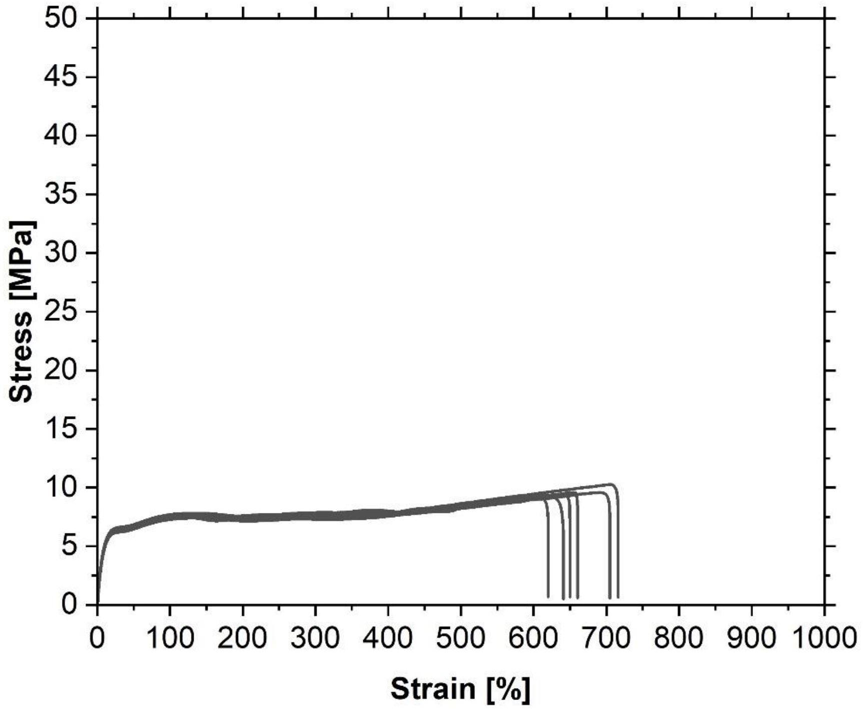

Figure S32: Stress-Strain curves of commercial LDPE (n=6).

Table S7: Tensile test data for tensile elongation tests of commercial LDPE (n=6).

entry	Stress at max. Force [MPa]	Strain at Breaking Point [%]	Young´s Modulus [GPa]
**1**	10.3	720	0.11
**2**	9.6	700	0.14
**3**	9.6	660	0.11
**4**	9.4	650	0.11
**5**	9.2	640	0.11
**6**	9.2	620	0.12
**average**	9.6	670	0.12
**standard deviation**	0.37	35	0.01

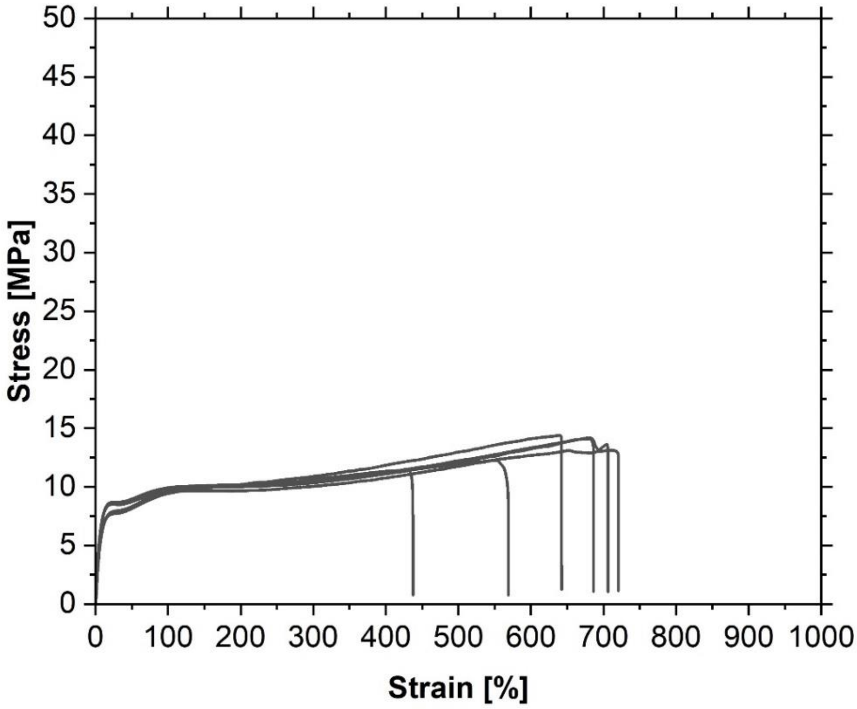

Figure S33: Stress-Strain curves of LDPE mimic **P4** (n=6).

Table S8: Tensile test data for tensile elongation tests of **P4** (n=6).

entry	Stress at max. Force [MPa]	Strain at Breaking Point [%]	Young´s Modulus [GPa]
**1**	13.1	720	0.21
**2**	14,2	710	0.16
**3**	14.1	690	0.17
**4**	12.3	570	0.16
**5**	14.4	640	0.08
**6**	11.4	440	0.18
**average**	13.3	630	0.16
**standard deviation**	1.10	98	0.04

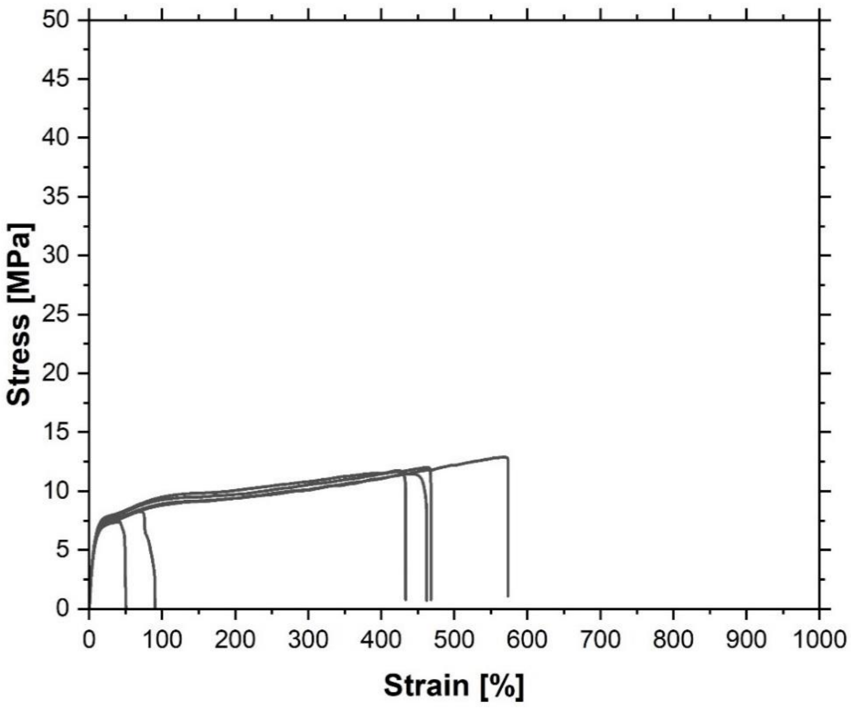

Figure S34: Stress-Strain curves of recycled LDPE mimic **P4** (n=6).

Table S9: Tensile test data for tensile elongation tests of **P4** (n=6).

entry	Stress at max. Force [MPa]	Strain at Breaking Point [%]	Young´s Modulus [GPa]
**1**	11.8	430	0.11
**2**	12.0	470	0.13
**3**	11.5	570	0.16
**4**	12.9	460	0.15
**5**	7.4	45	0.10
**6**	8.3	75	0.18
**average**	10.7	340	0.14
**standard deviation**	2.0	200	0.03

Density Measurements

Table S10: Density calculation of commercial LDPE (n=5).

entry	Density [g/cm^3]
1	0.919
2	0.929
3	0.915
4	0.922
5	0.918
average	0.921
standard deviation	0.005

Table S11: Density calculation of LDPE mimic **P4** (n=5).

entry	Density [g/cm^3]
1	0.9399
2	0.9395
3	0.9321
4	0.9273
5	0.9330
average	0.934
standard deviation	0.004

Rheology Measurements

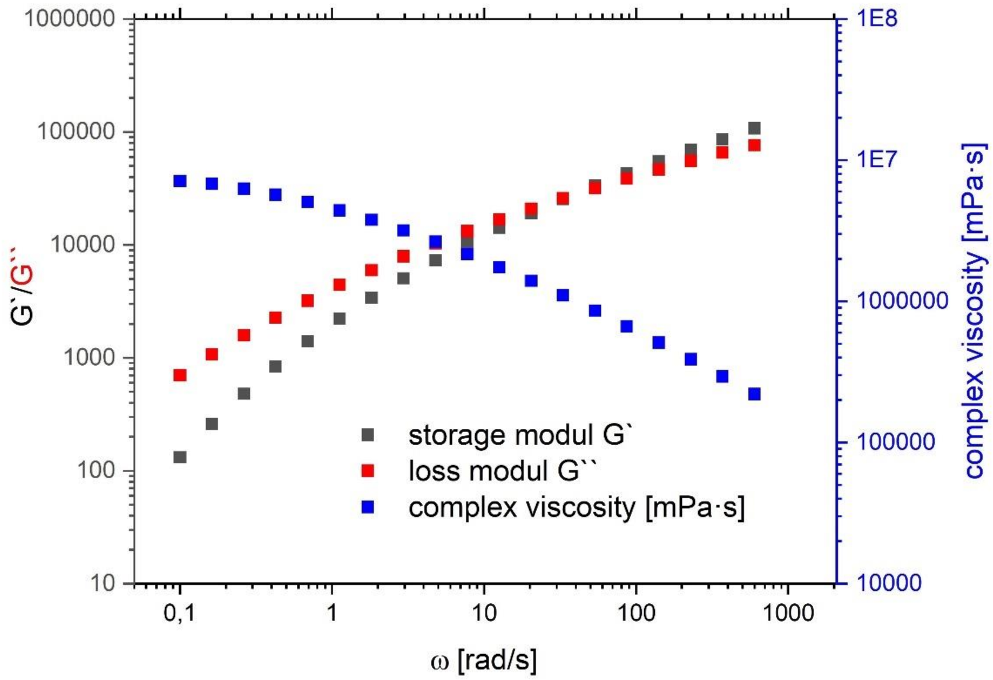

Figure S35: Frequency sweep of commercial LDPE at 130 °C.

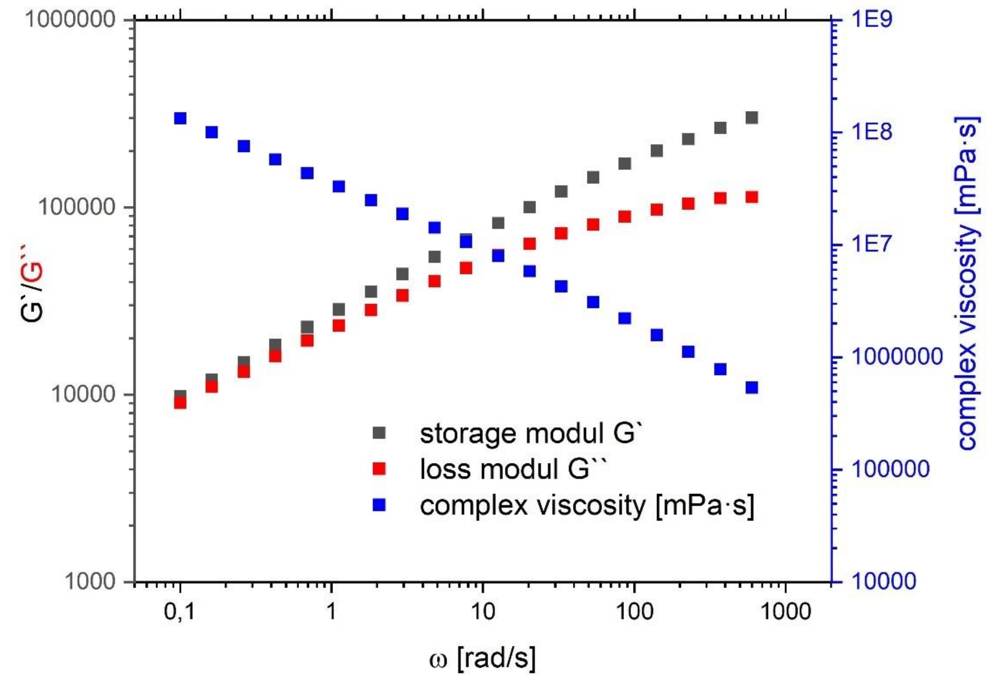

Figure S36: Frequency sweep of LDPE mimic **P4** at 130 °C.

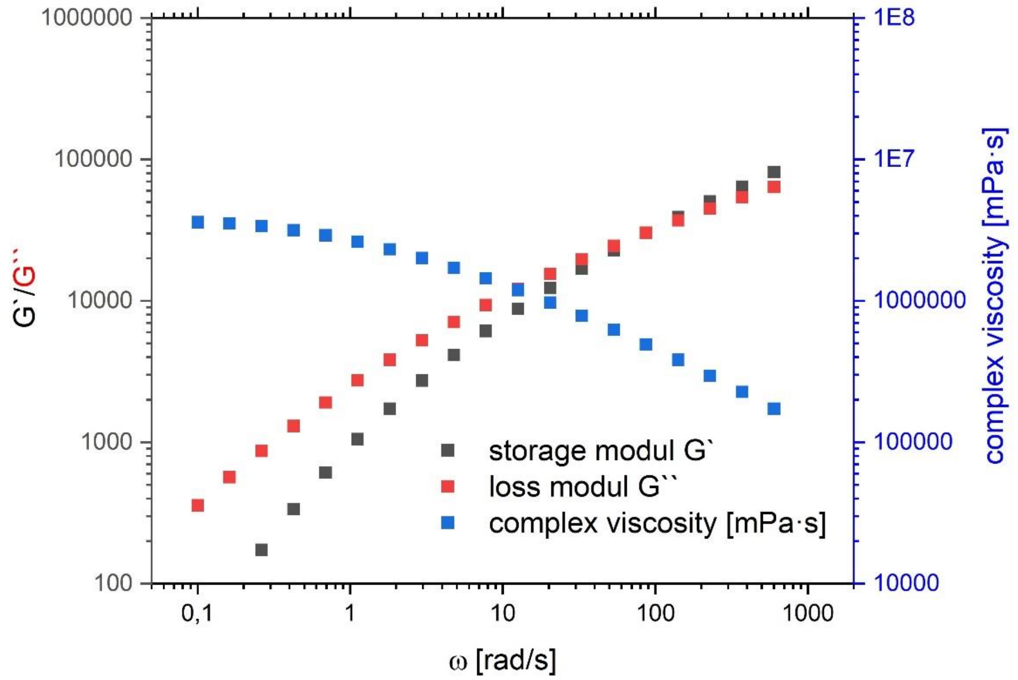

Figure S37: Frequency sweep of commercial LDPE at 150 °C.

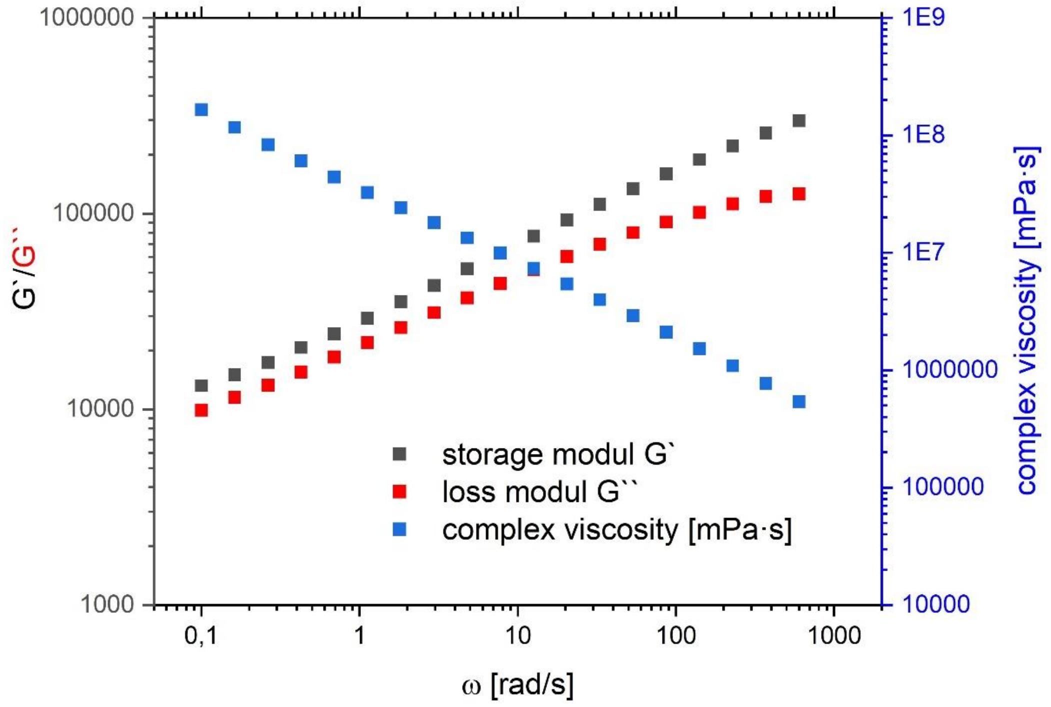

Figure S38: Frequency sweep of LDPE mimic **P4** at 150 °C.

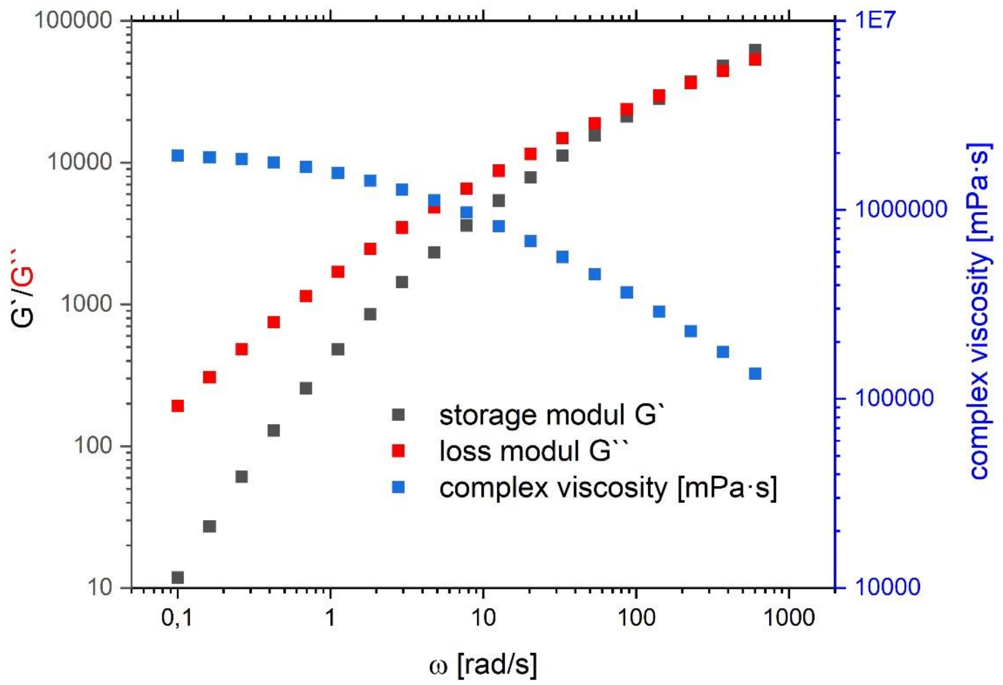

Figure S39: Frequency sweep of commercial LDPE at 170 °C.

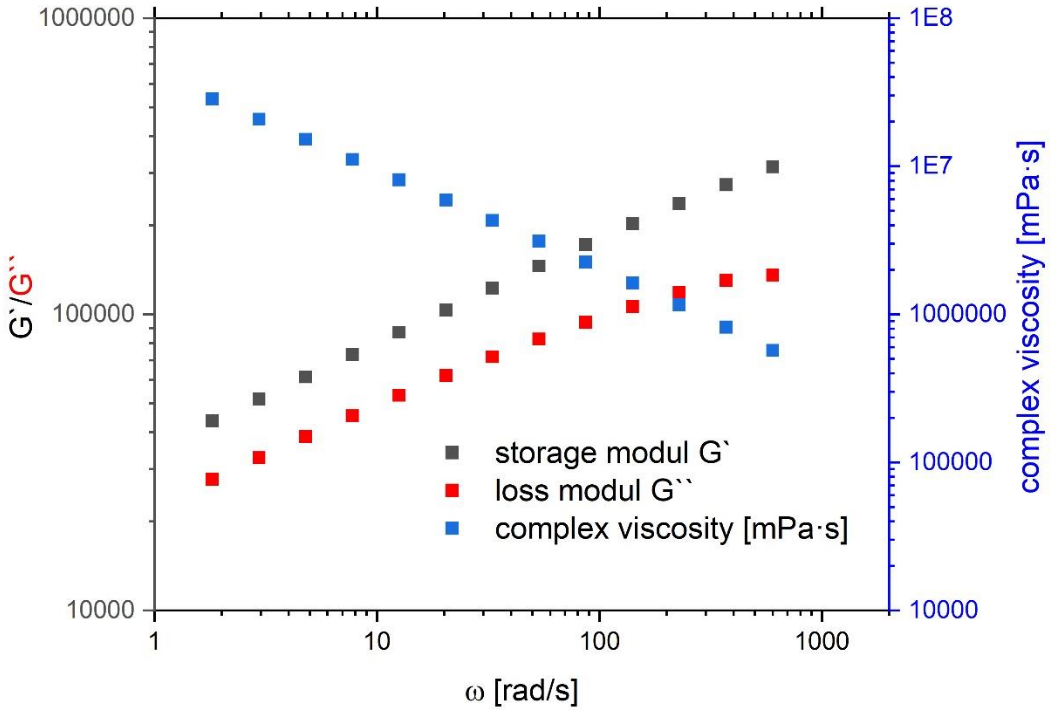

Figure S40: Frequency sweep of LDPE mimic **P4** at 170 °C.

Solubility Experiments

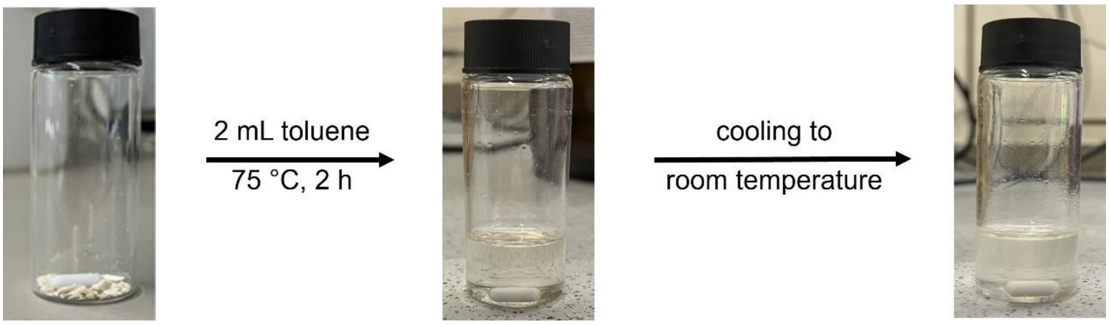

Figure S41: Solubility of the degrafted LDPE mimic **P4** in toluene.

For solubility experiments 30 mg of the degrafted LDPE mimic **P4** was mixed with 2 mL toluene. After heating to 75 °C and stirring for two hours a clear polymer solution was observed, which indicates that the low molecular weight polymer fragments yielded by degrafting of the LDPE mimic **P4** are well soluble in organic solvents at moderate temperatures (T=75 °C).

References

[1] S. K. T. Pillai, W. P. Kretschmer, M. Trebbin, S. Förster, R. Kempe, *Chem. Eur. J.* **2012**, *18*, 13974-13978.
[2] A. Noor, *Crystals* **2021**, *11*, 886.
[3] E. V. Avtomonov, K. A. Rufanov, *Z. Naturforsch.* **1999**, *54*, 1563-156.
[4] K. Shinohara, M. Yanagisawa, Y. Makida, *Sci. Rep.* **2019**, *9*, 9791.
[5] J. Martin, *J. Appl. Polym. Sci.* **1990**, *40*, 1801-1803.
